# Differences in vocal brain areas and astrocytes between the house wren and the rufous-tailed hummingbird

**DOI:** 10.3389/fnana.2024.1339308

**Published:** 2024-03-27

**Authors:** Carolina López-Murillo, Santiago Hinestroza-Morales, Pablo Henny, Jorge Toledo, Gloria Patricia Cardona-Gómez, Héctor Rivera-Gutiérrez, Rafael Posada-Duque

**Affiliations:** ^1^Área de Neurofisiología Celular, Grupo de Neurociencias de Antioquia, Instituto de Biología, Facultad de Ciencias Exactas y Naturales, Universidad de Antioquia, Medellin, Colombia; ^2^Laboratorio de Neuroanatomía, Departamento de Anatomía, and Centro Interdisciplinario de Neurociencia, NeuroUC, Escuela de Medicina, Pontificia Universidad Católica de Chile, Santiago, Chile; ^3^Scientific Equipment Network REDECA, Faculty of Medicine, University of Chile, Santiago, Chile; ^4^Área de Neurobiología Celular y Molecular, Grupo de Neurociencias de Antioquia, Facultad de Medicina, Sede de Investigaciones Universitarias, Universidad de Antioquia, Medellin, Colombia; ^5^Grupo de Investigación de Ecología y Evolución de Vertebrados, Instituto de Biología, Universidad de Antioquia, Medellin, Colombia

**Keywords:** vocal areas, HVC, LMAN, RA, astrocytes, GFAP, hummingbird, songbird

## Abstract

The house wren shows complex song, and the rufous-tailed hummingbird has a simple song. The location of vocal brain areas supports the song’s complexity; however, these still need to be studied. The astrocytic population in songbirds appears to be associated with change in vocal control nuclei; however, astrocytic distribution and morphology have not been described in these species. Consequently, we compared the distribution and volume of the vocal brain areas: HVC, RA, Area X, and LMAN, cell density, and the morphology of astrocytes in the house wren and the rufous-tailed hummingbird. Individuals of the two species were collected, and their brains were analyzed using serial Nissl- NeuN- and MAP2-stained tissue scanner imaging, followed by 3D reconstructions of the vocal areas; and GFAP and S100β astrocytes were analyzed in both species. We found that vocal areas were located close to the cerebral midline in the house wren and a more lateralized position in the rufous-tailed hummingbird. The LMAN occupied a larger volume in the rufous-tailed hummingbird, while the RA and HVC were larger in the house wren. While Area X showed higher cell density in the house wren than the rufous-tailed hummingbird, the LMAN showed a higher density in the rufous-tailed hummingbird. In the house wren, GFAP astrocytes in the same bregma where the vocal areas were located were observed at the laminar edge of the pallium (LEP) and in the vascular region, as well as in vocal motor relay regions in the pallidum and mesencephalon. In contrast, GFAP astrocytes were found in LEP, but not in the pallidum and mesencephalon in hummingbirds. Finally, when comparing GFAP astrocytes in the LEP region of both species, house wren astrocytes exhibited significantly more complex morphology than those of the rufous-tailed hummingbird. These findings suggest a difference in the location and cellular density of vocal circuits, as well as morphology of GFAP astrocytes between the house wren and the rufous-tailed hummingbird.

## Highlights

- This comparative study shows a differential spatial localization of vocal areas between the house wren and rufous-tailed hummingbird.
- The LMAN of the rufous-tailed hummingbird was more prominent and had higher cell density, while Area X was shown to be higher cell density in the house wren.
- GFAP astrocytes were more abundant in the house wren compared to the rufous-tailed hummingbird.
- GFAP astrocytes from the pallium of house wren exhibited greater morphology complexity than the rufous-tailed hummingbird.

## Introduction

Vocalization in birds is a complex behavior essential to finding mates, defending territory, and maintaining social cohesion (Beecher and Brenowitz, 2005; Mooney et al., 2007). Some birds learn and memorize songs composed of sequences of various notes with different frequency and amplitude patterns over time, with variations in repertoire between species ranging from complex (many songs and compositions) to simple songs (few songs and notes) (Brainard and Doupe, 2002; Araya and Wright, 2013). Vocal learning in birds has been detected in three different groups: hummingbirds (Apodiformes), parrots (Psittaciformes), and songbirds (Passeriformes, Oscines) (Jiménez et al., 2001; Scott, 2005; Mooney, 2009a). Although parrots and songbirds are phylogenetically related groups, hummingbirds do not share the same evolutionary history (Skutch, 1931; Baptista and Schuchmann, 1990; Williams and Houtman, 2008; Araya and Wright, 2013). Therefore, vocal learning may have had an independent evolutionary origin in these three groups.

The learning and production of song in birds involves a neural pathway connecting different vocal brain regions (Fortune and Margoliash, 1992; Smith, 1996) and the transmission of signals to the syrinx nuclei and respiratory pathways (Brainard and Doupe, 2002; Mooney, 2009a), thereby facilitating the production of song (Bradbury and Vehrencamp, 2011; Roberts and Mooney, 2013; Giordani et al., 2018). The bird’s brain has a nuclear organization consisting of regions such as the pallium, striatum, and pallidum (Jarvis et al., 2005; Pfenning et al., 2014). Hummingbirds and songbirds belong to two groups of birds that have standard forebrain circuits, involved in song production (Gahr, 2000); parts of this circuit are organized as nuclear-like structures. Neuroanatomical differences in the vocal brain areas of common tropical species that may serve as model species, such as the house wren and the rufous-tailed hummingbird, have yet to be described.

The neural circuit involved in acquiring and producing a learned song is composed of several interconnected brain regions that form three main pathways. The Song Motor Pathway (SMP), controls song production and primarily involves the high vocal center (HVC) located in the posterior nidopallium and the robust nucleus of the arcopallium (RA). The Anterior Forebrain Pathway (AFP) mediates song learning and plasticity and is mainly composed of Area X in the striatum and the lateral magnocellular nucleus of the anterior nidopallium (LMAN) (Sibley and Ahlquist, 1990; Araya and Wright, 2013). These two pathways converge with a third pathway, the auditory pathway, allowing perception of songs from the same species, different species, and even from the same individual (Nottebohm et al., 1976; Jarvis et al., 2000). The three pathways converge in the HVC nucleus, which plays a fundamental role in song learning and production (Mello, 2004; Reiner et al., 2004b). It has been observed that vocal areas such as the HVC, RA, and Area X show significant variations in size and shape, even within the same order of birds, and that these differences are closely related to the breeding season (Scharff and Nottebohm, 1991; Smith, 1996; Gulledge and Deviche, 1997; Smith et al., 1997; Brenowitz et al., 1998; Soma et al., 1999; Gahr, 2000; Poirier et al., 2008; Hall et al., 2010; Vellema et al., 2010, 2011; Fitch and Jams, 2015). These morphological variations between species and orders are pertinent to understanding brain organization concerning to song regions and their impact on the specific vocal capabilities of each species.

Song type and an individual’s learning stage can induce changes in the neural connections between different areas that, in turn, affect neuron properties. During the breeding season, there is an increase in recruited neurons in the HVC, mainly from the ventricular zone and olfactory bulb, suggesting a higher abundance during reproductive periods (Nieto, 2003; Kopec and Carew, 2013). However, it is essential to recognize the role of glial cells in ensuring the proper functioning of the central nervous system, such as the involvement of astrocytes in maintaining cerebral homeostasis and regulating synaptic transmission and neuronal plasticity in the vocal brain circuits (Tramontin and Brenowitz, 2000; Duncan and Saldanha, 2011; Haim and Rowitch, 2016; Zhang et al., 2016; Allen and Eroglu, 2017; Bailey et al., 2017). Although there are similarities in the organization of vocal areas between the different orders of birds (Fitch and Jams, 2015), differences have been documented in both the types of cells involved in vocal production and their distribution in the learning areas (Jarvis et al., 2000). A detailed study of this variation in the distribution and apparition of the vocal areas allows us to deepen our understanding of the neuroanatomical basis of vocalization in different wild bird species (Soma et al., 1999).

Astrocytes constitute a remarkably diverse cell population, demonstrating distinct morphologies, molecular profiles, anatomical distributions, physiologies, and functions across various species (Falcone, 2022). Different astrocyte markers such as GFAP and S100β exist. GFAP (Glial Fibrillary Acidic Protein) is considered a prototypical marker that provides information about the shape and function of astrocytes. This structural protein of the cytoskeleton plays a role in intracellular transport and is related to the integrity of the neurovascular unit (Wynne et al., 2008; Duncan et al., 2013). Regarding astrocytic protein S100β, it plays an important role in regulating intracellular calcium levels in astrocytes, influencing calcium homeostasis in the brain and, consequently, synaptic transmission (W’mningham-Major et al., 1989; Michetti et al., 2019). Nevertheless, most research on astrocytes has focused primarily on mammals and rodents, which are highly complex and heterogeneous, possibly due to the complexity of the central nervous system and its energy demands (Miller and Raff, 1984; Oberheim et al., 2009; Verkhratsky et al., 2019; Falcone, 2022). Astrocyte morphology and functions in birds are like those of mammals (Falcone, 2022). During the breeding season, an increase in the number and complexity of GFAP astrocytes has been observed in the HVC area of the canary *Serinus canaria* (Kafitz et al., 1999). Furthermore, vimentin astrocytes are prominent in the juvenile stage and in the fall, with a simpler morphology compared to GFAP astrocytes (Nottebohm et al., 1986; Kafitz et al., 1999; Kubikova et al., 2014). It has been observed that species with larger brains and more developed cognitive abilities have a higher density and complexity of astrocytes (Diamond et al., 1985; Colombo et al., 2006; Eroglu et al., 2009; Oberheim et al., 2009; Jeff and Cagla, 2017). These investigations suggest that the appearance of astrocytes contributes to increased brain size and behavior complexity (Kálman and Pritz, 2001; Falcone, 2022) it necessary to clarify the role of astrocytes in volitional communication, which is considered a complex behavior that occurs in birds as song learning.

Astrocytes appear to modulate vocal learning and production circuits, a sexually selected trait in many bird species (Gadagkar et al., 2016; Turk et al., 2021). There are few studies characterizing astrocytes in vocal areas, which have been conducted on migratory birds and non-passerine birds (Steinman et al., 2013; Carvalho-Paulo et al., 2018; Karatu et al., 2020). In mammals, astrocytes participate in dopaminergic circuits and motor control, which suggests that these glial cells have an essential role in the modulation of vocal production circuits, which is a sexual characteristic in many bird species (Gadagkar et al., 2016; Turk et al., 2021). Although astrocytes may play a role in somatosensory circuit refinement during postnatal stages (Molofsky et al., 2014), their involvement in adult birds has been poorly investigated.

**Figure 1:**
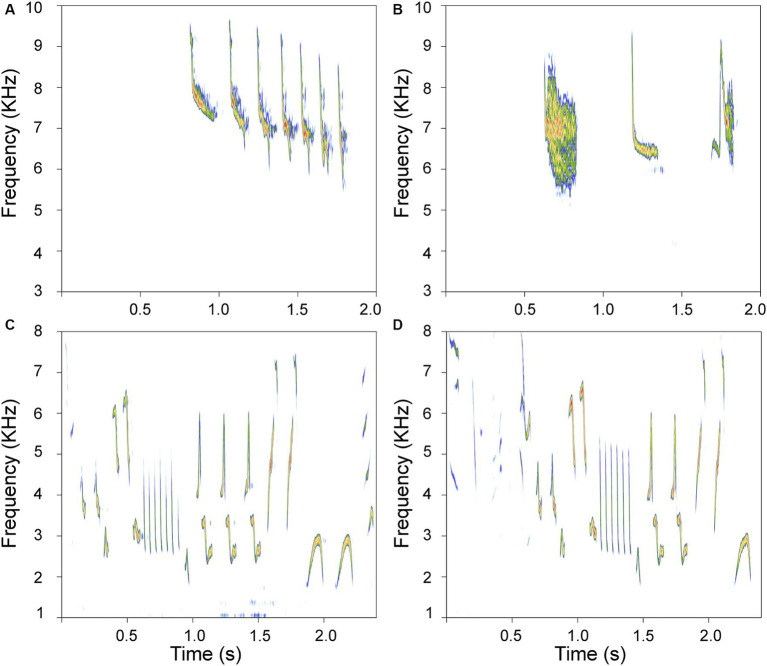
Examples of songs differing in complexity. **(A,B)** are sonograms of simple songs produced by the rufous-tailed hummingbird; **(C,D)** depict complex songs produced by the house wren. These spectrograms show representative motifs recorded from a house wren and available recordings of adult Rufous-tailed hummingbirds at www.Xeno-Canto.org (XC821348, XC843613). Each motif was taken from a different song. Note that each rendition is very different for each species.

Despite the distant phylogenetic relationship between hummingbirds and songbirds, there is evidence of convergent learned singing in these species (Gahr, 2000; Jarvis et al., 2000; Araya and Wright, 2013; Johnson and Clark, 2020; Kuhl et al., 2021). The functionality of hummingbird singing is equivalent to that of songbirds, and this character is generally considered ancestral and widespread but only sporadically present (Monte et al., 2023). Notably, many tropical hummingbirds coexisting with songbirds have remarkable songs (Araya and Wright, 2013) that vary in complexity. Bird species produce songs with varying temporal and structural complexity (Clive, 2008; Bradbury and Vehrencamp, 2011). While the definition of acoustic complexity is still debated (Mikula et al., 2018), the number of different acoustic elements produced, known as repertoire size, is considered a reliable measure of complexity and provides biologically relevant information (Clive, 2008). Bird species can exhibit simple (formed by few acoustic elements) or complex (many different acoustic elements) repertoires. While songbirds (Passeriformes, suborder Passeri) produce and learn complex songs, hummingbirds are known for their simpler vocalizations. Two well-characterized species in Colombia exemplify this contrast: the house wren (Passerine oscine: *Troglodytes aedon* (*Ta*)) produces complex songs with many different elements and with high variation in frequency and temporal characteristics. On the other hand, there are hummingbirds such as the rufous-tailed hummingbird [Trochiliforme: *Amazilia tzacatl* (*At*)], which produce songs with few different acoustic elements and with low variation in acoustic structure. Examples of such varied acoustic complexity are depicted in Figure 1.

Animal behavior is not solely driven by neuronal activity but involves coordinated interactions between astrocytes and neurons (Kofuji and Araque, 2021). Understanding the role of astrocytes in vocal brain circuits is crucial for a comprehensive understanding of how behaviors originate and can be complex. Since the astrocytes are critically involved in the control of complex motor behaviors, such as vocal circuits (Khan et al., 2001; Morquette et al., 2015; Xin et al., 2019; Corkrum et al., 2020; Turk et al., 2021; Turk and SheikhBahaei, 2022), and these species differing song complexities; we hypothesized differences in brain vocal areas, such as more relative size, cell density, and a more complex astrocyte structure and organization in the house wren compared to a rufous-tailed hummingbird. Also, since these species frequently perch and cohabit rural and semi-urban landscapes along with their ease of observation in tropical regions, we conducted a study comparing the distribution of the HVC, RA, Area X, and LMAN areas, cell density, and astrocyte morphology in these wild species.

## Materials and methods

### Bird information

Male individuals of the house wren (*Ta*) (*n* = 9) and the rufous-tailed hummingbird (*At*) (*n* = 4) were collected at the University of Antioquia – Campus Sede Oriente, Carmen de Viboral. The same sampling effort was made for both species in two reproductive seasons; however, collecting house wren was much easier due to their territorial behavior and abundance. Juvenile and female birds were avoided to preserve their population index and to discount any neurobiological differences due to age. These species are considered common and abundant, classified as “Least Concern” in Colombia’s birds red list (Echeverry-Galvis et al., 2022). Additionally, they are widely distributed and relatively common in both urban areas and agricultural landscapes. Furthermore, these species have a broad distribution and are relatively housed in urban areas and agroscapes (Scott, 2005). The collection of biological specimens is covered by the framework permit for the collection of wild specimens for non-commercial purposes, which was issued by ANLA in resolution 1,461 of December 3, 2014. The size sampling permission and the procedure was approved by the Ethics Committee for Animal Experimentation at the University (CEEA), resolution 139 of March 29, 2021.

### Wild bird collection

Birds were captured using the “*Manual of Methods for Biodiversity Inventory Development*” (Álvarez et al., 2004). They were collected using four 12 × 2 m mist nets with 30 mm mesh eye nets, each checked every 20 min from 6:00 to 13:00 h. Captured individuals were identified as sacrificed, and various morphometric parameters were measured, including sex, mass, exposed and total culmen length, beak height and width, closed wing length, and tarsus length. For euthanasia, isoflurane was administered at a dose of 2 mL per 125 g B.W. or less in a sealed box containing a cotton ball soaked with the anesthetic. The box was allowed to saturate with the anesthetic for at least 3 min, and then the bird was placed in the anesthesia chamber, the lid was closed, and the birds were left undisturbed for 30–60 s until they became immobile. If they remained active after this time, the dose was re-administered. After the last breath of the bird, a 30-s waiting period was observed, then decapitation was performed, and the skull was dissected to extract the brain. During the procedure, a portion of the metatarsus, breast, liver, and gonads were also collected for pathological analyzes and hormonal status. The brain of one individual of the house wren was not extracted but was preserved in a 4% paraformaldehyde solution, along with the bony part of the head. Table 1 depicts the collected individuals’ information.

**Table 1:** Data of collected birds.

Individual	Weight (g)	Exposed Culmen (mm)	Total Culmen (mm)	Beak Width (mm)	Beak Height (mm)	Tarsus Length (mm)	Closed Wing Chord (mm)	Sex
Ta1	16.70	14.4	10.2	4.5	4.7	13.1	60	Male
Ta2	17.72	12.8	15.6	4.4	4.2	13.8	54	Male
Ta3	15.98	12.7	16.6	4.8	4.0	–	60	Male
Ta4	15.33	13.5	15.7	5.7	4.0	23.5	55	Male
Ta5	14.47	12.6	17.5	15.3	3.1	12.0	55	Male
Ta6	15.29	12.1	18.1	14.2	3.9	13.4	55	Male
Ta7	–	12.4	17.5	4.7	3.8	21.7	56	Male
Ta8	15.96	12.1	18.5	4.5	4.7	12.4	50.7	Male
Ta9	15.08	12.8	19.5	8.1	3.5	22.0	54	Female
At1	5.06	21.3	25	3.9	2.4	4.0	57.2	Male
At2	4.66	20.5	23.4	4.0	3.0	4.7	55	Male
At3	4.06	21.2	24.7	3.3	3.0	4.2	53.6	Male
At4	–	21.1	–	4.3	2.9	–	53	Male

From every individual, the weight (g), the total and exposed culmen (mm), the width and height of the beak (mm), the length of the metatarsus (mm), and the closed wing chord (mm), as well as the sex. *Ta*, *Troglodytes aedon* – House wren; *At*, *Amazilia tzacatl* – *Rufous tailed hummingbird*.

After extracting the brain, it was washed with PBS 1X solution and then fixed in 4% paraformaldehyde in a cytoskeleton buffer with changes every 24 h for 3 days (Posada-Duque et al., 2013). Subsequently, a sucrose gradient was performed with concentrations of 7, 25, and 30% over three consecutive days, increasing the concentration daily. The brains that were not used immediately were stored at −20°C in a cryopreservative solution; the rest were sectioned using a cryostat.

### Tissue sectioning

One brain was selected for each of the two species. Once the sucrose gradient process was completed, the brains were washed with phosphate Buffer (PB) (0.1 M pH: 7.4) and a sagittal cut of the hemispheres was made using a blade. Then, using a 0.5 mm needle, four lateral-to-medial perforations were made along each hemisphere. This was done to subsequently locate the perforations in the slices and align the tissues for the 3D reconstruction of the main vocal areas. Next, parasagittal sections of the entire hemisphere were prepared using the cryostat (LEICA CM1850 UV). Each hemisphere embedded in OCT was positioned laterally on the specimen holder, with a weight on top, and allowed to freeze (approximately 3–5 min). Then, the entire hemisphere was sliced using a blade (LEICA 819 - Low Profile) with blade changes every 20 cuts, and the sections stored in 48-well plates with PB 0.1 M solution (pH: 7.4) + 0.1% Azide. Later, the sections were mounted on labeled glass slides (SuperFrost PLUS-0006E) in the correct order and left to dry in a dust-protected tray.

### Nissl staining for the 3D reconstruction of vocal brain areas

A gradual rehydration was performed by slightly tilting each slide to quickly impregnate the tissues with 100, 70, and 50% alcohol. Then, excess alcohol was removed, through rinsing with distilled water, and allowed to drain. Next, the tissues were stained with Toluidine Blue dye [1:10] and left to act, followed by rinsing with distilled water and draining. Subsequently, dehydration was carried out using 70, 96, and 100% alcohol. The final dehydration and clearing were done in an extraction chamber using xylene, and Shandon Consul-Mount (Thermo Scientific; 9,990,440) was added to adhere the glass cover, and then the slides were left to dry.

### Whole brain imaging, processing, and analysis

All the Nissls slides were scanned with a NanoZoomer-XR brightfield tissue scanner microscope (Hamamatsu), equipped with a 20x objective (NA 0.75; UPlanSApo; Olympus) using a 40x digital zoom at a single layer. They were opened with QuPath software (Bankhead et al., 2017) to perform quality control (excluding tissues with tears or folds) and export each tissue as TIFF format, considering the order of each tissue for subsequent analysis with Stereo Investigator software (MBF Bioscience - MicrobrightField, Version: 2021). One hemisphere from each species was selected to draw and obtain the 3D reconstruction of the vocal areas and perform volumetric quantification using Neurolucida Explorer software (MBF Bioscience - MicrobrightField, Version: 2021).

For the delimitation and reconstruction of the vocal areas, the following parameters were considered: *i.* A detailed review of the research by Karten et al. (2013) and Stokes et al. (1974) for the house wren, and the research by Jarvis et al. (2000) and Gahr (2000) for the rufous-tailed hummingbird, in order to locate the vocal areas and identify the parameters they had used. *ii.* The sequential order of the tissues for each species. *Iii.* Histological description of the shape of each area, cell labeling, and cell clustering; type of cellular labeling that distinguishes the center or interior from the border or exterior of each area. *iv.* Sequential and complete drawing of each of the vocal areas. *v.* Location of markers (icons) on the perforations made with the needle for 3D rotation and reconstruction. *vi.* Rotation of the tissues to fit into the tracings and markers made at each hole created with the needle. *Vii.* Validation of the vocal areas in a different individual from the 3D reconstruction using immunohistochemistry for the nuclear marker mouse anti-NeuN (Sigma-Aldrich; MAB377; [1:250]) and with mouse anti-MAP2 (Sigma-Aldrich; M9942; [1:250]), using different tissues that included the initial and final bregmas of the main areas of the song (Supplementary Figure S1A).

### Mediolateral location of vocal nuclei

After recognizing the anatomical vocal brain nucleus, its most medial and lateral limits (in μm from midline) were determined. To compare the location and width of a given song nucleus between both species, the nucleus’s relative mediolateral limits for each species were calculated, considering 0 as the midline and 1 as the brain maximal extension. The song nuclei’s maximal width was calculated by subtracting each nucleus’s most lateral and medial limits.

### Analysis of the cellular density profile in the vocal areas

From the Nissl-stained tissues used for 3D reconstruction, 50% of the bregmas encompassing each of the four vocal areas were selected, and cell counting was performed. Cellular density analysis was conducted using a fractionated scanning method with equidistant grids to count cells in the area of interest. Cell counts were performed within these grid points and then extrapolated to the entire area of interest. The “Area Fraction Fractionator” tool in the Stereo Investigator software allowed for the estimation of cell counts within an area of interest using predetermined parameters for grid size and distance between grid points, which were then extrapolated to the total measured area (Supplementary Figure S1B). Using the “icon” option, each cell within the grid was marked without touching the lower or left boundary for each grid point in the entire area of interest. At the end of this process, the cell counts were extrapolated to the size of the total area, ensuring that the Cruz-Orive/Geiser, Schmitz-Hof, and Scheaffer error coefficients were below 0.1 to verify the accuracy of the sampling.

Images scanned from Nissl stained slices within the vocal brain area per specimen were processed and analyzed using QuPath software (Bankhead et al., 2017). Whole nuclei were delineated, and cells per 1 mm^2^ from three slices were quantified from whole delineated nuclei of the house wren (*n* = 7) and rufous-tailed hummingbird (*n* = 4).

### Immunohistochemistry

Sections of 30 μm were obtained using a cryostat, similar to those used for the 3D reconstruction of vocal brain areas. Antigen retrieval was performed using 1X citrate buffer (pH: 6) (Master-diagnostic; MAD-004071R/D) at 85°C for 20 min with constant dripping onto the tissues. Subsequently, endogenous peroxidase activity was blocked with a peroxidase blocker (Master-diagnostic; MAD-021540Q-125) for 20 min to prevent nonspecific antibody binding. Samples were incubated in 1% bovine serum albumin (BSA; Sigma-Aldrich, A9647), 0.3% Triton-X100 (Sigma-Aldrich, T9284-500ML), and 0.1 M PB (pH: 7.4) for 1 h at room temperature with constant agitation. This was followed by a 72-h incubation at 4°C in a primary antibody solution containing anti-GFAP mouse (Sigma-Aldrich; G3893 – RRID: AB_477010; [1:250]), anti-S100β rabbit (Dako-Agilet; Z0311 – RRID: AB_10013383; [1:250]), anti-MAP2 mouse (Sigma-Aldrich; M9942 – RRID: AB_477256; [1:250]), or anti-NeuN mouse (Sigma-Aldrich; MAB377 – RRID: AB_2298772; [1:250]) diluted in 0.3% BSA, 0.3% Triton-X100, and 0.1 M PB with constant agitation. After 20 min of washing off excess antibodies, the tissues were incubated for 2 h at room temperature with biotinylated goat anti-mouse secondary antibody (Invitrogen; 31,800 – RRID: AB_228305; [1:250]) or biotinylated goat anti-rabbit secondary antibody (Invitrogen; B-2770 – RRID: AB_2536431; [1:250]) diluted in 0.3% BSA, 0.3% Triton-X100, and 0.1 M PB with constant agitation. Subsequently, the tissues were vigorously washed in 0.1 M PB solution three times for 5 min each, followed by a 1-h incubation in the avidin-biotin peroxidase standard staining kit (Thermo Scientific; 32,020) at room temperature. Development was carried out for 3–4 min using 3,3′-diaminobenzidine tablets (Sigma-Aldrich; D4293). The tissues were then mounted on glass slides and subjected to sequential dehydration with ethanol (70, 96, 100%) and xylene. Finally, without allowing the tissues to dry with xylene, coverslips were mounted using Shandon Consul-Mount (Thermo Scientific; 9,990,440) for drying and observation under the microscope.

### Imaging of immunohistochemistry

Whole brain imaging for GFAP slides [*Ta* (*n* = 6) *At* (*n* = 4)] was scanned with a Ventana-DP 200 brightfield tissue scanner microscope (Roche), equipped with a 40x objective (NA 0.75; UPlanSApo; Olympus) using a 40x digital zoom at a single layer.

Unique cell imaging for fractal morphological analysis of astrocytes was performed using brightfield microscope imaging. For this purpose, five cells were recorded per telencephalic region in the pallium (Laminar edge of pallium (LEP) and vascular pallium), the pallidum, and the mesencephalon for *Ta* (*n* = 6) and *At* (*n* = 3). These cells were randomly selected and captured using an Olympus CX35 microscope (Model X31RBSFA) equipped with a 100x oil immersion objective (NA 1.25; PlanC N; Olympus) and a Swift camera (SC1803R).

### Bright-field image analysis

Image processing of Nissl and GFAP was performed in QuPath 0.3.2 software for *Ta* (*n* = 6) and *At* (*n* = 4) (Bankhead et al., 2017). Quantifying the percentage of GFAP area in pallium was performed using a particle detector using the same threshold, and the GFAP area was shown by heatmap.

The single-cell images were processed, segmented, and analyzed using FIJI software (NIH ImageJ) (Schindelin et al., 2012). For the morphological analysis of astrocytes, the images were converted to 8-bits, and astrocyte somas were manually segmented using the brush selection tools. The Simple Neurite Tracer plugin was utilized to segment the cellular processes, and then a merge of the soma and processes of each astrocyte was obtained (Longair et al., 2011). Subsequently, morphological parameters of the soma, cellular processes, and the entire cell were obtained using the FracLac plugin (Karperien, n.d.) and FIJI tools. Finally, based on Fernández-Arjona et al. (2017), morphological characteristics related to complexity (fractal dimension, lacunarity, roughness, density, and compactness), size (area, perimeter, and major axes), shape (circularity and aspect ratio), and spatial domain (scaling and shape features of the convex hull, bounding rectangle, and fitted ellipse) were analyzed.

### Immunofluorescence of brain samples

The brains of songbirds were extracted and fixed as previously described. Subsequently, the samples were sectioned into parasagittal slices of 30 μm thickness using a Leica cryostat for songbirds. Before immunostaining, antigen retrieval was performed on the songbird slices using citrate buffer at 95°C for 20 min, respectively. Autofluorescence was blocked using 50 mM NH_4_Cl prepared in H_2_O. The samples were incubated in 1% bovine serum albumin (BSA, Sigma-Aldrich, A9647) for 1 h at room temperature to prevent nonspecific antibody binding. The brain sections were incubated for 72 h at 4°C in primary antibodies, rabbit anti-GFAP (Sigma-Aldrich; ab5804 – RRID: AB_305124; [1:250]) with mouse anti-NeuN (Sigma-Aldrich; MAB377 – RRID: AB_2298772; [1:250]), diluted in an antibody solution containing 0.3% BSA, 0.3% Triton-X100, and PB (0.1 M, pH 7.4). After removing excess antibodies through a 20-min wash, the sections were incubated for 2 h at room temperature in a secondary antibody solution with goat anti-mouse Alexa Fluor 488 (Invitrogen; A-11001; 1:250) and goat anti-rabbit Alexa Fluor 594 (Invitrogen; A-11012; [1:250]); the nuclear marker Hoescht (Vector Labs; DL-1068; [1:2500]) was also incubated. Subsequently, the samples were vigorously washed in PB (0.1 M) three times for 5 min each. Finally, the sections were mounted on glass slides with FluorSave Reagent (Millipore; 345,789).

### Confocal microscopy of birdsong brain samples

Astrocytes in the telencephalon and mesencephalon for bird song were captured using confocal microscopy. Three high-magnification images per slide were obtained from the triple immunostaining, and they were captured using an Olympus FV1000 scanning confocal microscope equipped with a 60X oil immersion objective (NA 1.42; PLAPON; Olympus) with a zoom factor of 4, three lasers at 488, 594 nm, and DAPI in FluoView 3.1.1.9 software (Olympus). Sixteen-bit TIFF images of 1,024 × 1,024 pixels (105.47 × 105.47 μm) were obtained with an XY pixel size of 103 nm and a 400 nm spacing between Z sections. 26 optical sections were captured from each field (with a thickness of 10 μm). In addition, we created a confocal mosaic image for each bird to generate a complete representation of the brain slide in GFAP areas, using a 10X air objective (NA 0.4; APLANPOS; Olympus). For the house wren, we used a 9×9 grid, while for the hummingbird, we used a 7 × 7 grid, and the final reconstructed images were achieved using FluoView 3.1.1.9 software (Olympus).

### Confocal image processing and analysis of birdsong brain samples

The confocal images were deconvoluted, processed, segmented, and 3D reconstructions were created. Image deconvolution was performed using Huygens Essential 23.3 software (Scientific Volume Imaging B.V.). The classic maximum likelihood estimation (CMLE) algorithm was used for image deconvolution with a signal-to-noise ratio of 21, and the bird images were deconvoluted using the deconvolution assistant. The images were converted to 8 bits and subsequently processed and analyzed in FIJI software. Immunofluorescence signals were segmented using intensity thresholding with the Huang algorithm to standardize fluorescence signals across all images. Z-projections using the standard deviation of segmented stacks were employed to assess astrocyte structure. These Z-projections were segmented and used to quantify astrocyte processes. Finally, for illustrative purposes, surface rendering was performed to display the 3D projections of deconvoluted images using the 3D Surface rendering option in Huygens Essential 23.4 (Scientific Volume Imaging B.V.).

### Sholl analyzes: number of astrocyte processes

The astrocyte processes were counted using deconvoluted images. Excess background illumination was subtracted from a value of 25 using the Image-Pro Plus Subtract tool. The images were processed, segmented, and analyzed using FIJI software (NIH ImageJ) (Schindelin et al., 2012). For crossing number of astrocyte processes, the images were converted to 8-bits and segmented using intensity thresholding by the Huang algorithm. Skeletonization was carried out, and the processes stemming from the soma were quantified using the Simple Neurite Tracer plugin, employing Sholl analysis to count the number of intersecting processes. Finally, the images were processed to obtain the representative figures.

### Statistical analysis

For the analysis of cell density in each of the vocal brain regions of the house wren (n = 7) and the rufous-tailed hummingbird (*n* = 4), cells were quantified in three sections, blind to the conditions. The GFAP area percentage was quantified in two sections, blind to the conditions. We performed a descriptive analysis of the distribution of each cell detected in the vocal areas through box and whisker plots, showing the median (middle line), 25th to 75th percentiles (box limits), and Min to Max value (whiskers limits) of house wren (green), and rufous-tailed hummingbird (orange), depicting cellular area quantifications performed in QuPath. Data normality was assessed using the Shapiro–Wilk test. A non-parametric Mann–Whitney test was conducted to compare cell density between the house wren and the rufous-tailed hummingbird.

Fractal morphology analysis and astrocyte intersections (Sholl analysis) were performed using five images per brain region for each specimen, house wren (*n* = 6) and hummingbird (*n* = 3). Data normality was assessed using the Shapiro–Wilk test. For parametric univariate data, a one-way analysis of variance (ANOVA) was employed, followed by Tukey’s multiple comparison tests to compare astrocytes within LEP, FLP, and FRL of the house wren. A non-parametric Mann–Whitney test was conducted to compare the fractal morphology and Sholl analysis of astrocytes in LEP between the house wren (*n* = 7) and the rufous-tailed hummingbird (*n* = 3). All groups were processed simultaneously to mitigate potential experimental variations. Statistical analyzes were performed using GraphPad Prism software, version 8.0. Significance levels were determined as follows: * for *p* < 0.05, ** for *p* < 0.01, and *** for *p* < 0.001.

## Results

### The vocal areas were closer to the midline in house wren, while they were intermediate in rufous-tailed hummingbird

Body condition reflects the quality of life related to survival, reproduction, and behavior. The wild birds collected during the breeding season and the morphometric patterns obtained were related to adult birds based on established criteria (Table 1) (Scott, 2005; Johnson and Clark, 2020). The brain volume-to-body weight ratio was 19,008 mm^3^/g for the house wren and 17,323 mm^3^/g for the rufous-tailed hummingbird.

**Figure 2:**
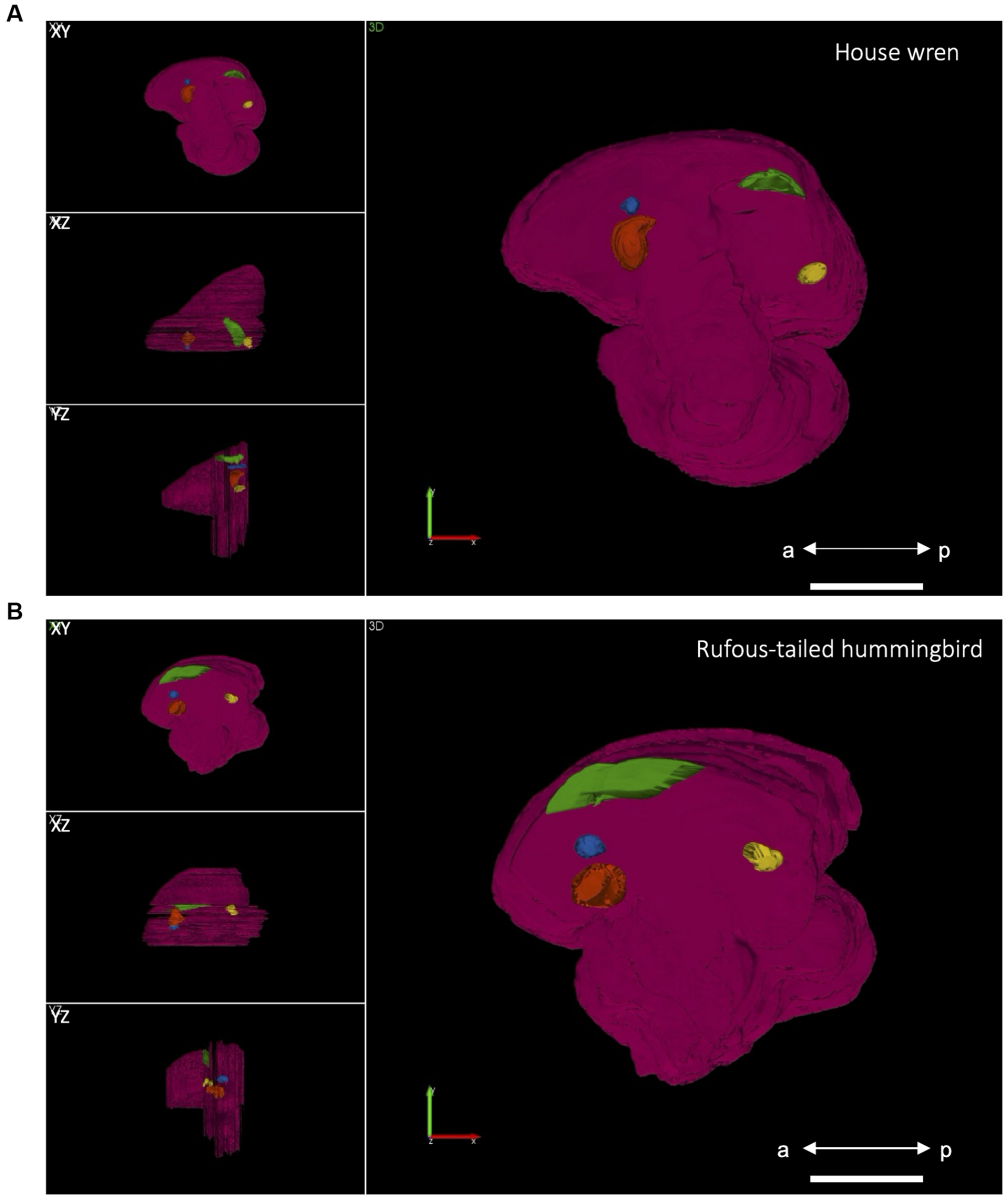
Three-dimensional reconstruction of the vocal brain areas in lateral (XY), ventral (XZ), and posterior (YZ) views. **(A)** In the house wren, the vocal brain areas are shown in a position close to the midline. Scale bar 3 mm. **(B)** In the rufous-tailed hummingbird, the vocal brain areas are shown in a position intermediate between the midline and the lateral aspect, and the HVC is located in an anterior position Scale bar 1.5 mm. The LMAN is shown in blue, Area X in orange, HVC in green, and RA in yellow; a: anterior position, p: posterior position.

Among bird species, there are variations in the location, shape, and volume of the vocal areas (Jarvis et al., 2000; Mooney, 2009b). From the manually delimited contours for the entire hemisphere and all the vocal areas, we generated a 3D brain reconstruction to describe the position and volume of representative vocal areas. In the house wren, the vocal areas were closer to the midline, whereas in the rufous-tailed hummingbird, they were at an intermediate location between the lateral portion and midline (Figure 2; Table 2). The house wren showed LMAN and Area X in an anterior hemisphere position, while RA and HVC were in a posterior location (Figure 2A; Supplementary Figures S2A,B; Table 2). Despite the rufous-tailed hummingbird showing similarity in the location of LMAN, Area X, and RA; the HVC area spans both the anterior-dorsal and posterior regions of the hemisphere, being longer but narrower compared to the house wren (Figure 2B; Supplementary Figures S2C,D). These findings suggest a differential spatial localization of vocal brain areas between the house wren and rufous-tailed hummingbird.

**Table 2:** Mediolateral location and width of telencephalic song nuclei.

	House wren				Rufous-tailed hummingbird			
	LMAN	Area X	RA	HVC	LMAN	Area X	RA	HVC
Brain mediolateral extension (μm)	0–7,140				0–3,900			
Nucleus mediolateral boundaries (μm)	(180–1,650)	(270–1,530)	(270–1,170)	(390–2,760)	(720–1,350)	(870–1890)	(1560–2070)	(1620–2070)
Nucleus relative mediolateral boundaries (0–1)	(0.03–0.23)	(0.04–0.21)	(0.04–0.16)	(0.05–0.39)	(0.18–0.35)	(0.22–0.48)	(0.40–0.53)	(0.42–0.53)
Nucleus relative width (0–1)	0.20	0.17	0.12	0.34	0.17	0.26	0.13	0.11

Size of the nuclei relative to the total mediolateral brain size. The relative widths were calculated from the relative mediolateral limit for each species (0–1).

Starting from the 3D construction of each nucleus, we determined the most medial and lateral limits and, hence, were able to calculate their mediolateral extensions (widths). Then, their widths were relativized to the maximal mediolateral extension of the hemisphere (Table 2). Interestingly, relative to the hemisphere extension, the house wren showed a wider HVC compared to the rufous-tailed hummingbird, while Area X was wider for the rufous-tailed hummingbird, compared to the house wren, suggesting differences in the size of production and learning vocal areas between both species.

### The volume and cellular morphology of vocal areas in house wren and rufous-tailed hummingbird

**Figure 3:**
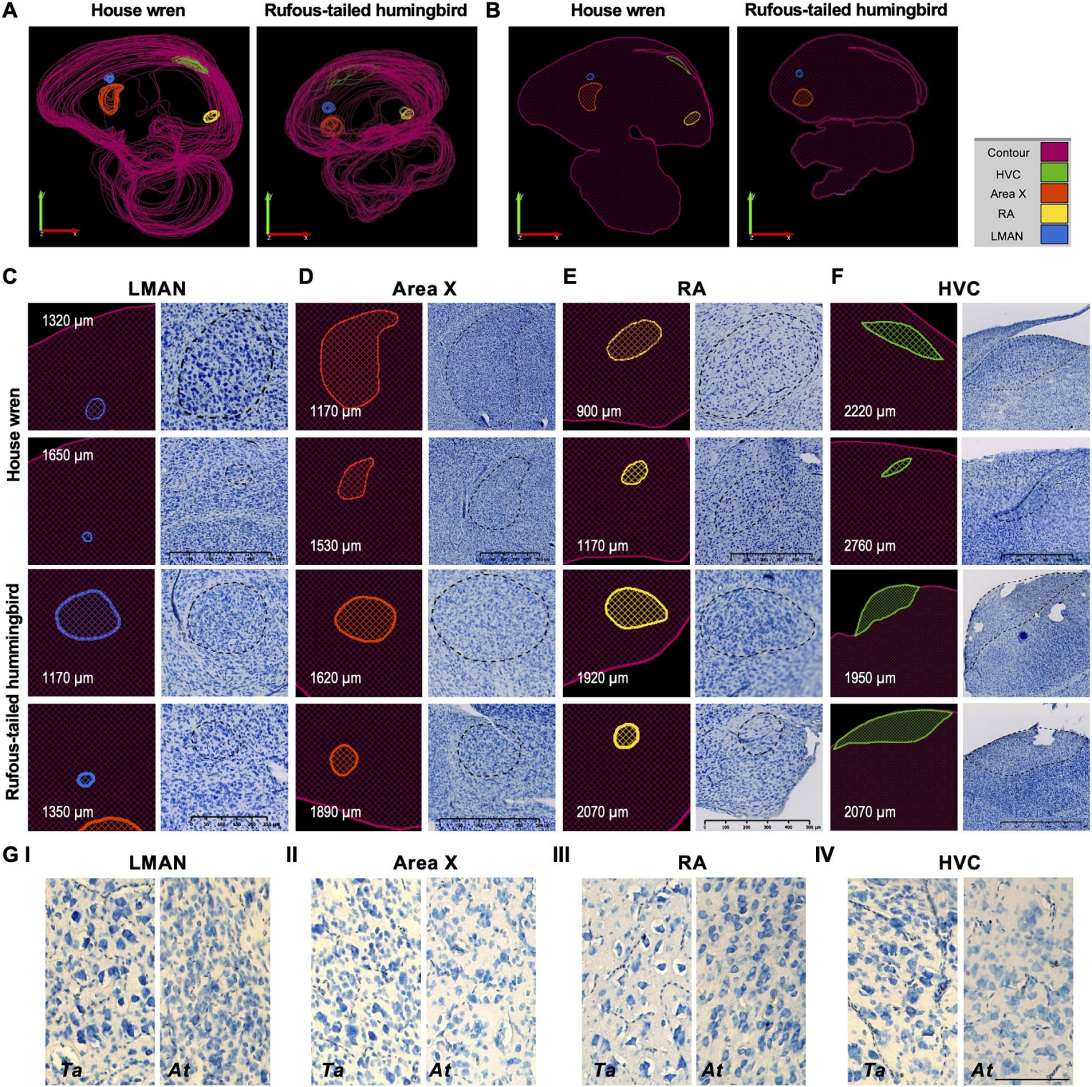
Reconstruction and localization of the vocal brain areas in the house wren and rufous-tailed hummingbird at different bregma levels from the midline to the lateral part. **(A)** Three-dimensional representation of the vocal brain areas of house wren and rufous-tailed hummingbird. The LMAN is shown in blue, Area X in orange, the HVC in green; and the RA in yellow. **(B)** Location of the vocal brain areas of house wren and rufous-tailed hummingbird, respectively. The value indicates mediolateral slices represented. The LMAN and Area X are shown in the anterior regions, while the HVC and RA are located in the posterior positions. **(C)** Traced contour (left) and image (right) are shown for all the vocal brain areas at two different mediolateral positions and for each species. The LMAN is shown at 1320 and 1,650 μm, and at 1170 and 1,350 μm from the midline in the house wren and rufous-tailed hummingbird, respectively. **(D)** The Area X is shown at 1170 and 1,530 μm, and at 1620 and 1890 μm from the midline in the house wren and rufous-tailed hummingbird, respectively. **(E)** The RA is shown at 900 and 1,170 μm, and at 1920 and 2070 μm from the midline in the house wren and rufous-tailed hummingbird, respectively. **(F)** The HVC is shown at 2220 and 2,760 μm, and at 1950 and 2070 μm from the midline in the house wren and rufous-tailed hummingbird, respectively. **(G)** Magnification of each of the vocal brain areas in both species. I LMAN; II Area X; III RA; IV HVC. Left side corresponds to house wren an right side corresponds to rufous-tailed hummingbird. **(C)** House wren: Scale bar: 500 μm, Rufous-tailed hummingbird: 250 μm, **(D,E)** Scale bar: 500 μm, **(F)** House wren: Scale bar: 500 μm, Rufous-tailed hummingbird: 1000 μm, **(G)** Scale bar: 100 μm.

**Figure 4:**
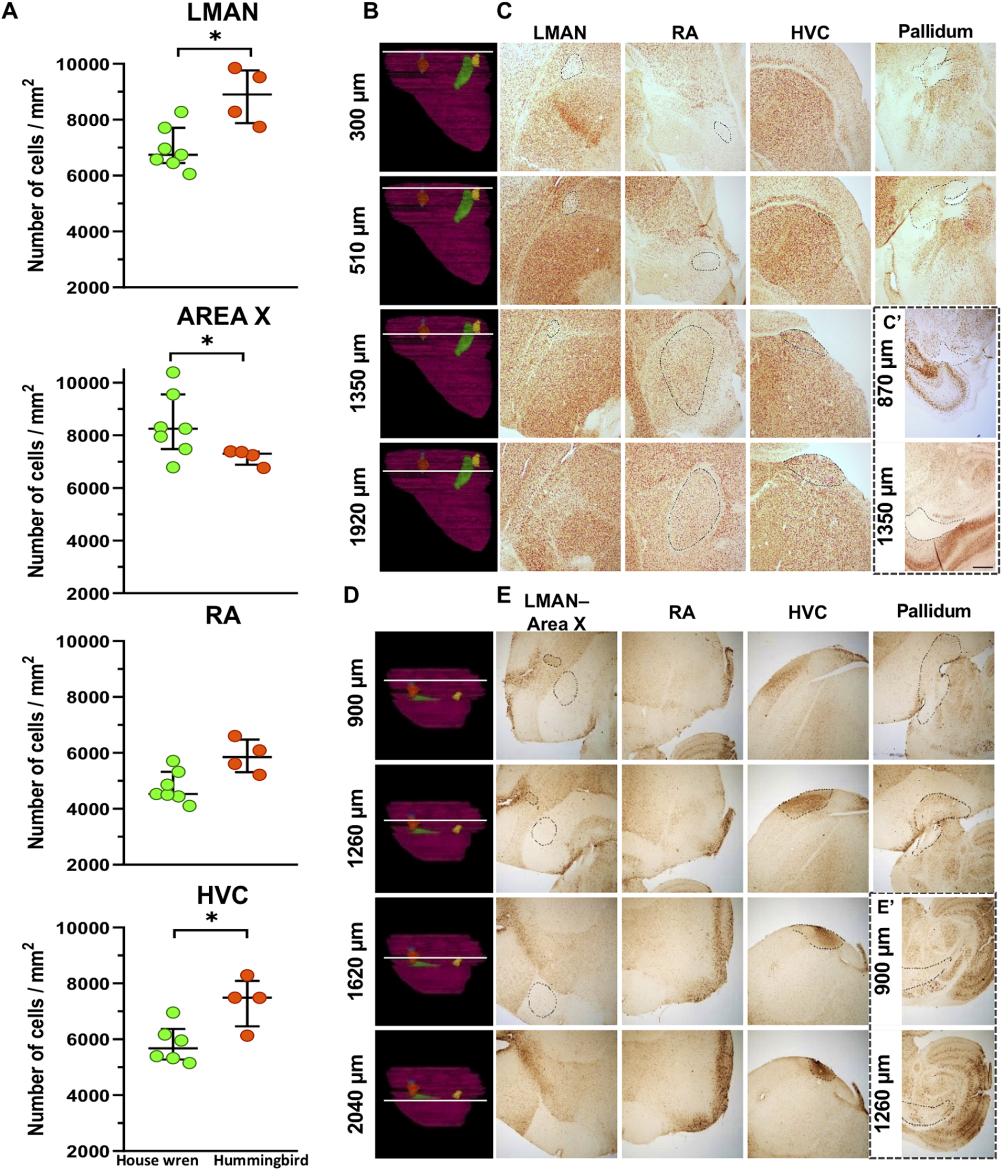
Cell density and validation at different bregma’s from the midline of the vocal brain areas in the house wren and rufous-tailed hummingbird using NeuN staining. **(A)** Comparison of cellular density in vocal areas among two species. Green circles show the house wren, and Orange circles show the rufous-tailed hummingbird. A representative diagram showing the location of each bregma across the width of the hemisphere in the house is shown in **(B,C)**. **(B)** The white line shows the location of each bregma. **(C)** The LMAN was validated from bregma 300 to approximately 1,350 μm, the RA area from bregma 300 to approximately 1920 μm, and the HVC area from 510 to approximately 1920 μm. **(C′)** Section showing the pallidum and mesencephalon where the white matter appears. Representative diagram showing the location of each bregma across the width of the hemisphere in the rufous-tailed hummingbird in **(D,E)**. **(D)** The white line shows the location of each bregma. **(E)** Validation of the LMAN located from bregma 900 to approximately 1,260 μm, the Area X from bregma 900 to approximately 1,620 μm, the HVC area from 1,260 to approximately 2040 μm. **(E′)** Section the pallidum and mesencephalon where the white matter appears. Statistical analysis was performed with Mann Whitney test for comparing distributions of two independent groups LMAN: * *p =* 0.0121; Area X: * *p =* 0.0424, RA: *p =* 0.0524 and HVC: * *p* = 0.0381. *Ta*: *n* = 7 – *At*: n = 4. **(C,D)** Scale bar: 500 μm.

We described the cellular morphology and distribution of vocal areas for the house wren and the rufous-tailed hummingbird based on whole and unique cellular imaging of the vocal areas and the quantitative area registration of each cell contained in each region (Figure 3). In the house wren and the rufous-tailed hummingbird, the LMAN area showed a volume of 0.0993 and 0.2018 mm^3^, respectively (Figure 3C). We observed large and elongated cells with a pyramid shape and intense staining, surrounded by scattered cells forming a sphere in the house wren; contrastingly in the rufous-tailed hummingbird, the LMAN area showed smaller elongated cells, closely packed together, surrounded by scattered cells forming an oval shape (Figures 3C,G.I; Supplementary Figure S3A). Interestingly, the LMAN of the rufous-tailed hummingbird showed an intense patch of blue color, suggesting a possible higher cellular density compared to the house wren (Figure 4A; Supplementary Figure S3A).

Area X had a volume of 0.6764 and 0.0405 mm^3^ in the house wren and the rufous-tailed hummingbird, respectively (Figure 3D). In the house wren, Area X showed various types of cells, including small, round, elongated, and big cells dispersed throughout the area, with intense-staining pyramid-shaped cells. Its shape resembled a water droplet, narrower at the upper dorsal and rounder at the lower ventral part. While in the rufous-tailed hummingbird, Area X was spherical and composed of cells of various sizes, with a predominance of big cells in clusters (Figures 3D,G.II; Supplementary Figure S3A).

The RA area in the house wren and the rufous-tailed hummingbird had volumes of 0.1694 and 0.0432 mm^3^, respectively (Figure 3E). In the house wren, the RA area was mainly comprised of pyramid-shaped cells spaced apart, with intense staining distinguishing them from the surrounding environment. An oval shape on the area’s outer part was formed by elongated and closely spaced cells. In contrast, in the rufous-tailed hummingbird, the RA area mainly showed round cells close to each other, with lighter staining compared to the elongated and separate surrounding cells, forming a slight oval shape around them (Figures 3E,G.III; Supplementary Figure S3A).

The HVC area showed 0.5431 and 0.1451 mm^3^ volumes for the house wren and the rufous-tailed hummingbird, respectively (Figure 3F). In the house wren, the HVC area displayed different types of cells distributed variably (Supplementary Figure S3A). Sections close to the midline showed small and closely packed cells forming a thin band near the hippocampus, while in sections further away from the midline, cells were a more dispersed and with a more intense staining. The HVC area took on an oval shape with flattened ends and was surrounded by small, elongated cells close to each other. And in the rufous-tailed hummingbird, the HVC area mainly exhibited round cells close to each other. At the upper-dorsal part of the area, elongated cells with intense staining were found, extending towards the anterior nidopallium, making its morphology similar to that of the house wren (Figures 3F,G.IV).

### Higher cell density in LMAN and Area X are, respectively, observed in the rufous-tailed hummingbird and the house wren

We performed the 3D rendering of each hemisphere and each vocal area and calculated the percentage of occupancy of each nucleus relative to the total size of the hemisphere. We analyzed the relation between the volume of the vocal areas and the total volume of the hemisphere in the house wren and the rufous-tailed hummingbird, and the LMAN showed an occupancy percentage of 0.0655 and 0.0924; Area X of 0.4459 and 0.4605; RA of 0.1117 and 0.0986; and HVC of 0.3580 and 0.3311, respectively (Table 3).

**Table 3:** Percentage of occupancy in songbird brain areas.

	House wren			Rufous-tailed hummingbird		
	Volume (mm^3^)	% Occupation in the hemisphere	% Cell density profile (# cells* μm^2^)	Volume (mm^3^)	% Occupation in the hemisphere	% Cell density profile (# cells* μm^2^)
LMAN	0.0993	0.0655	0.01032	0.0405	0.0924	0.02249
Area X	0.6764	0.4459	0.01716	0.2018	0.4605	0.01303
RA	0.1694	0.1117	0.00598	0.0432	0.0986	0.00832
HVC	0.5431	0.358	0.01089	0.1451	0.3311	0.01119
Hemisphere	151.6833			43.8265		

The data shows the occupancy percentage and cell density profile of LMAN, Area X, RA, and HVC in the house wren and rufous-tailed hummingbird.

In order to explain the volumetric differences between both species, we performed a cellular density analysis of the vocal areas. For this purpose, a fractionated scanning method was used in the area of interest, overlaying equidistant grids (as shown in Supplementary Figure S1B). Although the cellular density in RA area was similar for both species, we observed that the house wren exhibited a higher cellular density in Area X, while in the rufous-tailed hummingbird, this higher density was found in the LMAN and HVC areas (Table 3) (Figure 4A; Mann–Whitney test, * *p* value <0.05). This suggests a differential occupation and cellular density of LMAN, HVC and Area X between the house wren and rufous-tailed hummingbird.

### The vocal areas showed MAP2 and NeuN markers, validating 3D location and nuclear pattern

The contours of the vocal areas were delineated through a meticulous analysis of their location and cellular and histological description. We used specific neuronal proteins, NeuN and MAP2, to validate the location of the vocal areas. Specifically, we used representative slices at the bregma where each vocal song area began and ended. The corresponding contours for LMAN, Area X, RA, and HVC were drawn in the specific location using both markers (Figure 4; Supplementary Figures S3B–E). The nuclear pattern and the location of all the vocal areas were found between positions 270–1920 μm, from the midline, for the house wren (Figures 4B,C; Supplementary Figures S3B,C) and 870–2040 μm for the rufous-tailed hummingbird (Figures 4D,E; Supplementary Figures S3D,E). This nuclei pattern from neuronal markers reproduces the 3D location of the vocal areas at the proposed bregma in the house wren and the rufous-tailed hummingbird.

### The house wren has a greater abundance of GFAP astrocytes compared to the rufous-tailed hummingbird

**Figure 5:**
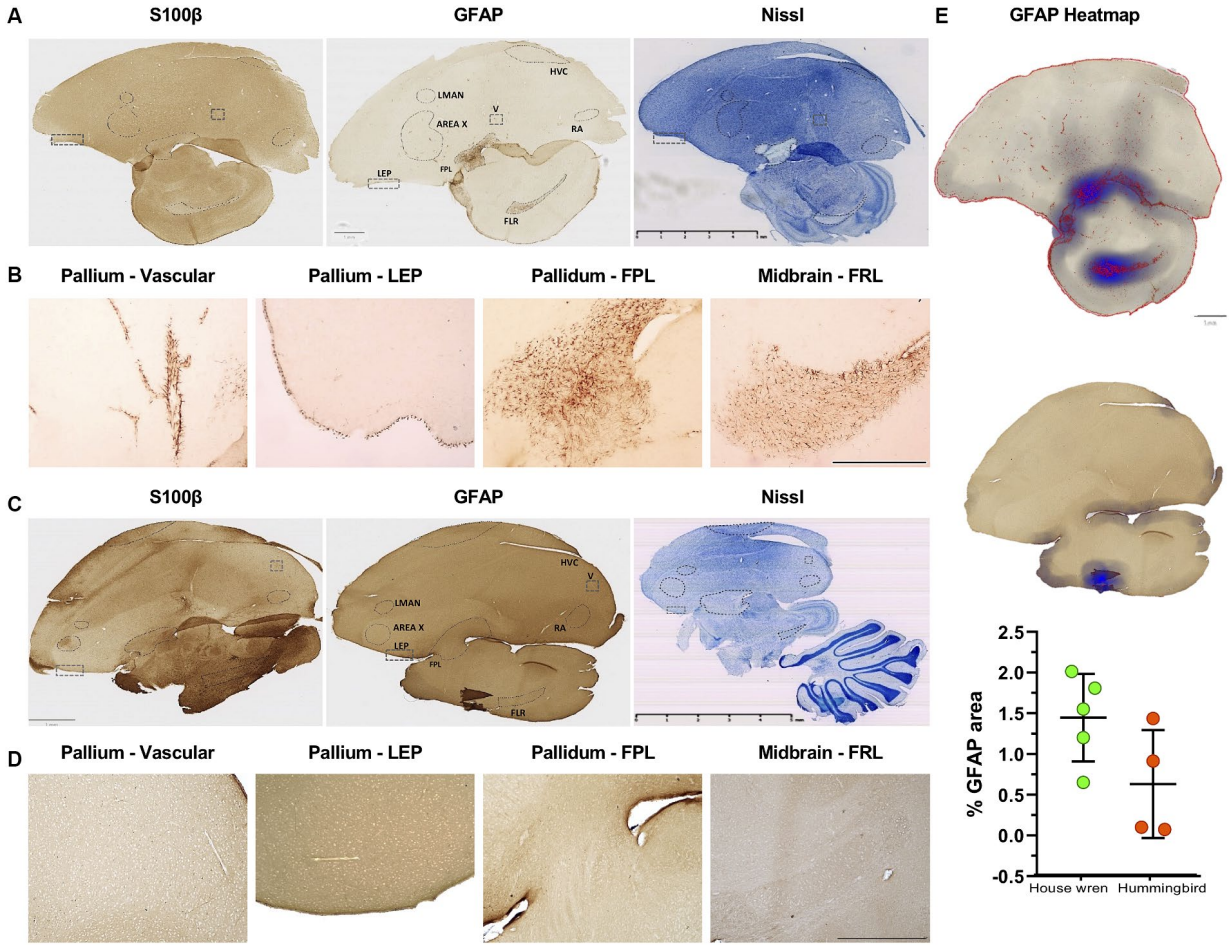
Different distribution of GFAP and S100 astrocytes in vocal and motor relay regions in the house wren and the rufous-tailed hummingbird. **(A)** Whole tissue scan with S100β, GFAP, and Nissl staining in the house wren **(B)** GFAP astrocyte labeling in the house wren brain regions such as the vascular pallium and the laminar edge of pallium (LEP), as well as in the pallidum in the FPL and the mesencephalon in FRL. **(C)** Whole tissue scan with S100β, GFAP, and Nissl staining in the rufous-tailed hummingbird **(D)** GFAP astrocyte labeling in the rufous-tailed hummingbird brain regions such as the vascular pallium and LEP, as well as in the pallidum in the FPL and the mesencephalon in the FRL. **(E)** Heatmap of GFAP staining of the pallidum and mesencephalon. Quantification of percentage of GFAP area in the pallium. Statistical analysis was performed with Mann Whitney test for comparing distributions of two independent groups, *p* value = 0.1111. GFAP astrocytes labeled in LEP region **(A,C)** Scale bar: 5 mm, **(B,D)** Scale bar: 500 μm. **(C–E)** Scale bar: 500 μm. *Ta*: *n* = 6 – *At*: *n* = 4.

**Figure 6:**
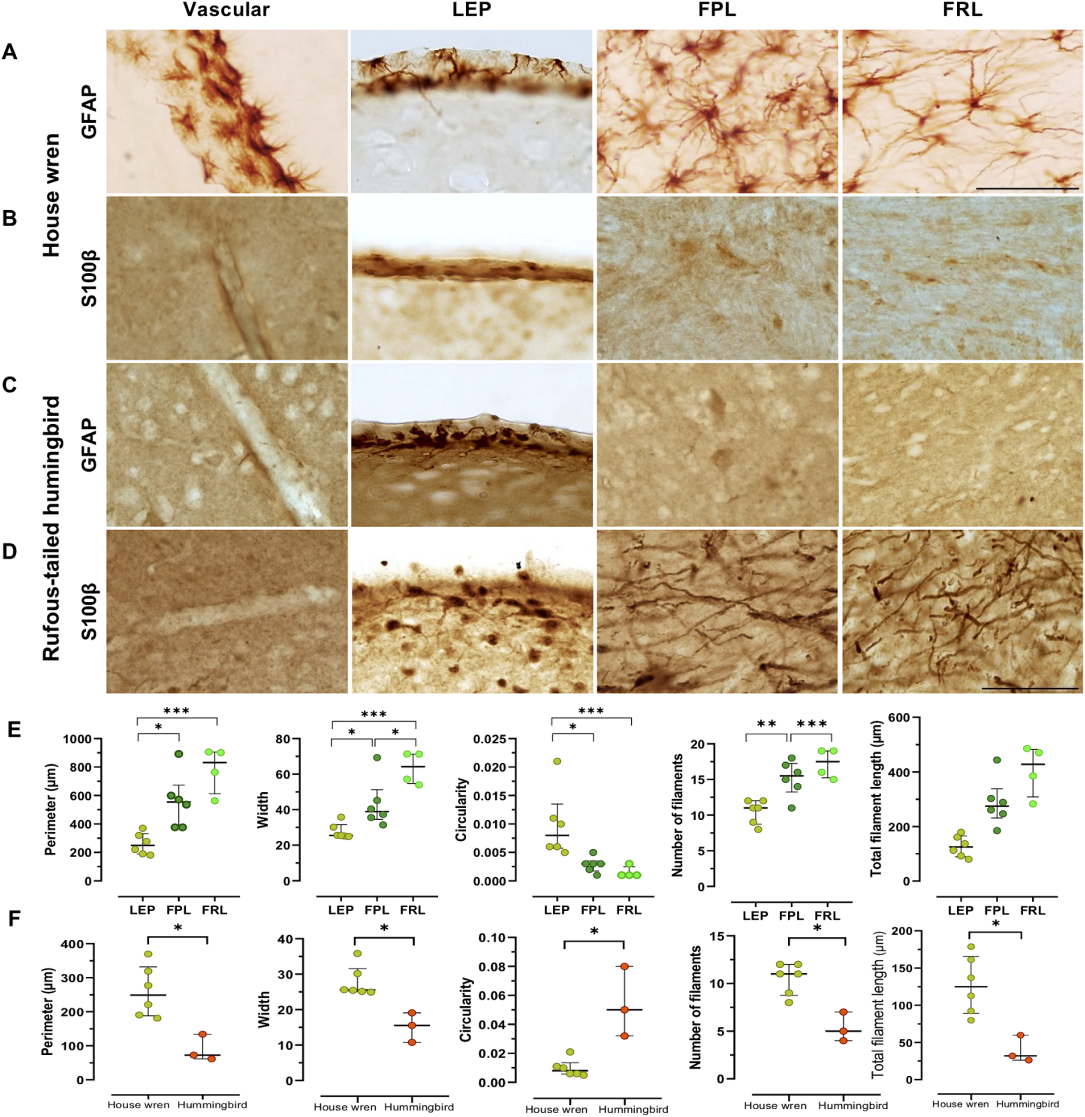
Morphology of GFAP and S100β astrocytes in the telencephalon of the house wren and the rufous-tailed hummingbird in the vascular pallium, LEP, FPL, and FRL. Representative images of GFAP astrocytes and S100β like processes of the house wren in **(A)** and **(B)**; and the rufous-tailed hummingbird in **(C,D)**, respectively. **(E)** Morphological parameters (perimeter, width, circularity, number of filaments, total filament length) of GFAP astrocytes processes in the house wren. **(F)** Comparison of morphological parameters of GFAP astrocytes processes in the LEP region between the two species. Green circles show the house wren, and orange circles show the rufous-tailed hummingbird. One-way ANOVA with Tukey’s *post hoc* test was performed for multiple comparisons for **(E)**. Mann Whitney test was performed to compare distributions of two independent groups for **(F)**. The significance levels were set at **(E)** Perimeter: * *p* = 0.0104, *** *p* = 0.0003; Width: * *p* = 0.0192, *** *p* = 0.0003, * *p* = 0.0458; Circularity: * *p* = 0.0190, * *p* = 0.0130; Number of filaments: ** *p* = 0.0052, *** *p* = 0.0007 and **(F)** Perimeter, Width, Circularity: * *p* = 0.0238; Number of filaments and Total filament length: * *p* = 0.0119. *Ta*: *n* = 6 – *At*: *n* = 3. **(A–D)** Scale bar: 50 μm.

Neuroanatomical studies of the vocal areas have focused on neuronal circuits (Gahr, 2000; Poirier et al., 2008; Vellema et al., 2011). However, astrocytes might participate in vocal areas (Kafitz et al., 1999; Haim and Rowitch, 2016). We labeled GFAP astrocytes with an antibody previously used in birds [Anti-GFAP (Rb) (Merk-millipore; ab5804)] (Polomova et al., 2019), and we also probed human and murine-tested anti-GFAP and anti-S100β antibody [anti-GFAP (Ms) (Sigma-Aldrich; G3893) and anti-S100β (Rb) (Dako-Agilet; Z0311)] (Rantamäki et al., 2013; Laddach et al., 2023). Consistently, we found that anti-GFAP tested for birds labeled effectively for both species, even for recognition in humans (Supplementary Figure S4A). Therefore, we examined the distribution of GFAP and S100β astrocytes in the vocal areas LMAN, Area X, RA, and HVC. Although these areas did not show classical astrocytic morphology, we found a GFAP and S100β punctate pattern in both species (Supplementary Figure S4B). Consistently, we found GFAP astrocytes in other brain regions, such as the telencephalon in the pallium located in the laminar edge of pallium (LEP) and vascular portions for house wren, as well as in the pallidum and mesencephalon, specifically, in the lateral prosencephalic fascicle (FPL) and the lateral mesencephalic reticular formation (FRL) for house wren (Figures 5, 6A,C). These regions were located at the same bregma point where the vocal areas were situated (Supplementary Figure S2), as was referenced in the known zebra finch atlas brain (Lovell et al., 2020). We did not observe an apparent label of S100β astrocytes in the same areas in the house wren (Figures 5A,B, 6A,B). In the rufous-tailed hummingbird, we found GFAP-positive astrocytes in the LEP region but not in the FPL and FRL, and consistently, we found a varicose pattern of s100β processes resembling astrocytes in the three regions (Figures 5C,B, 6C,D, and Supplementary Figure S5). We quantified the total GFAP in the pallium and mesencephalon and showed it on a heat scale for each species, finding that GFAP astrocytes were more abundant in the house wren than in the rufous-tailed hummingbird (Figure 5E).

### The GFAP astrocytes in the pallium of the house wren display more complex morphological features compared to those in the rufous-tailed hummingbird

The GFAP astrocytes of each area were segmented and analyzed for fractal parameters to determine their complexity. The house wren showed a typical morphology of GFAP astrocytes and cells with low levels of somatic S100β (Figures 5A,B). Comparing GFAP astrocytes within house wren, the GFAP astrocytes of the FPL and FRL showed a greater complexity, with more extended and more filamentous structures compared to astrocytes at LEP (Figures 6A,E, and Supplementary Figure S6A; ANOVA and Tukey’s test, * *p* < 0.05, ** *p* < 0.01, and *** *p* < 0.001). Interestingly, in the rufous-tailed hummingbird, S100β astrocytes exhibited a more straightforward punctate pattern in the LEP and a pattern of long filamentous in the FPL and FRL (Figure 5S). In addition, we found typical GFAP astrocytes in the LEP rufous-tailed hummingbird (Figure 5D). Therefore, we characterized the fractal morphology of GFAP astrocytes in the LEP of both species, resulting in a greater perimeter, width, and process length in the house wren compared to the rufous-tailed hummingbird (Figures 6A,C,F; Supplementary Figure S6B; ANOVA and Mann–Whitney test, * *p* < 0.05).

**Figure 7:**
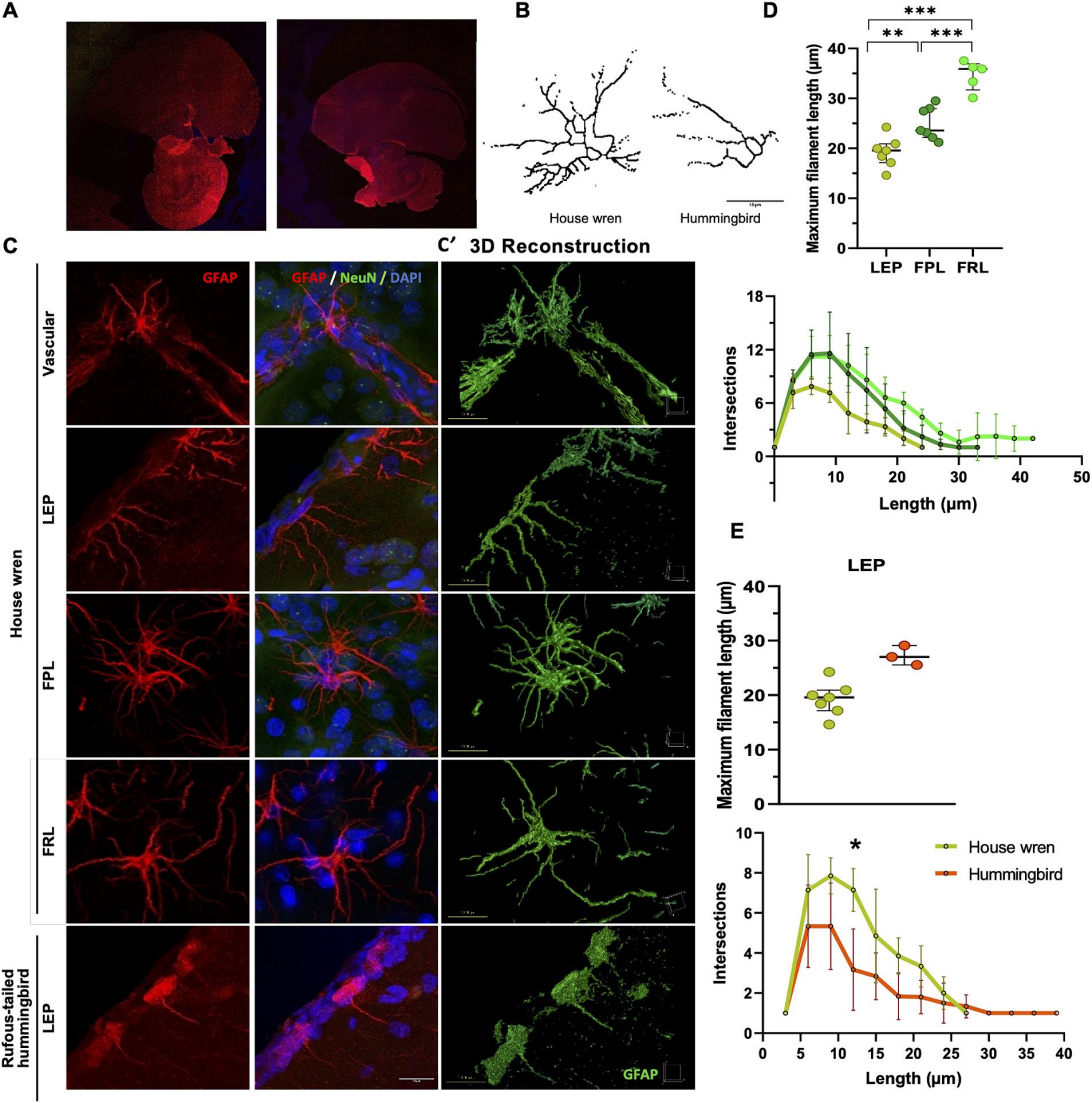
Comparison of processes ramification of GFAP astrocytes of the house wren and the rufous-tailed hummingbird. **(A)** Confocal tiling imaging of whole GFAP staining (red) in the house wren and the rufous-tailed hummingbird. **(B)** Representative images of GFAP astrocyte skeleton of sholl analysis from LEP of the house wren and hummingbird. **(C)** Representative 3D maximal projection images of GFAP astrocyte (red), NeuN (green), and nuclei (blue) immunofluorescence of the house wren (LEP, FPL, and FRL) and the rufous-tailed hummingbird (LEP). **(C′)** Representative surface 3D rendering images of GFAP astrocyte (green) of the house wren (LEP, FPL, and FRL) and the rufous-tailed hummingbird (LEP). **(D)** The maximum length and number of intersections of astrocytic processes in the LEP, FPL, and FRL of house wren. **(E)** Comparison of GFAP astrocytes in the LEP region between the two species. Green circles show the house wren, and orange circles show the rufous-tailed hummingbird. One-way ANOVA, and Tukey’s *post hoc* test were performed for multiple comparisons for **(D)**; and t-test comparison for **(E)**. The significance levels were set at **(D)** ** *p =* 0.0082, *** *p =* 0.0002 and **(E)** * *p =* 0.0105. *Ta*: *n* = 7 – *At*: *n* = 3 **(C)** Scale bar: 10 μm.

Subsequently, we performed a morphological analysis comparing GFAP astrocytes between the two species using immunofluorescence and confocal microscopy. This analysis would support observations by immunohistochemistry and delve into the three-dimensional detail of the astrocytes. Specifically, we identified astrocytes within the regions above (Figures 7A,B). Furthermore, the Sholl analysis, which shows the branching of astrocytic processes and GFAP astrocytes in the house wren, exhibited greater complexity across all examined areas, specifically in FPL and FRL, as shown above (Figures 7C,D; ANOVA and Tukey’s test, ** *p* < 0.01, and *** *p* < 0.001 7; 7E; Mann–Whitney test, * *p* < 0.05). Consistently, LEP GFAP astrocytes were compared; they showed more significant branching in the house wren compared to those in the rufous-tailed hummingbird. These findings indicate that astrocytes are considerably more complex in the house wren compared to the rufous-tailed hummingbird.

## Discussion

This comparative study introduces a novel framework for examining variations in the localization, volume, and cell density of vocal areas and disparities in astrocyte characteristics, which may support variations in the complexity of songs between these two wild bird species. This comparative study shows a differential spatial localization of the vocal areas of the house wren and the rufous-tailed hummingbird. Specifically, the LMAN of the rufous-tailed hummingbird was more prominent and had a higher cell density, while Area X was shown to have a higher cell density in the house wren. GFAP astrocytes were more abundant in the house wren compared to the rufous-tailed hummingbird, and LEP GFAP astrocytes in the house wren exhibited greater morphological complexity than the rufous-tailed hummingbird.

The study introduces a 3D reconstruction of vocal brain regions, LMAN, Area X, RA, and HVC, in the house wren and rufous-tailed hummingbird obtained through advanced microscopy and histological analysis, which facilitates volumetric and cellular analysis of the vocal brain areas. The provided volumetric, spatial, and cellular data is significant given the fact that no prior brain atlas existed for the song regions of these two species, allowing for comparison based on vocal complexity.

Specifically, these birds perch and coexist in rural and semiurban buildings, and they require a conspecific tutor to learn vocalization, like humans. Introducing a bioacoustic comparison, it is widely accepted that the house wren and the rufous-tailed hummingbird differ in the complexity of their songs, with the former having a complex song and the latter a simple one. Wrens (*Trogloditidae*), in general have a complex song (Kroodsma, 1980; Marler and Peters, 1988), and the song of the rufous-tailed hummingbird and similar species is characterized by simplicity and less variability in their repertoire (Mooney, 2009a; Jarvis, 2019).

Both species exhibited brain nucleus associated with vocal production and learning, along with GFAP astrocytes, indicating a convergent neural substrate for learning (Jarvis et al., 2000; Araya and Wright, 2013; Johnson and Clark, 2020; Kuhl et al., 2021). Recent findings demonstrate that hummingbirds have acquired songs functionally equivalent to those of songbirds, suggesting homology in vocal brain areas (Monte et al., 2023). This study uniquely establishes a relation between the convergence of GFAP astrocytes and regions involved in motor control (Morquette et al., 2015; Xin et al., 2019; Corkrum et al., 2020; Turk and SheikhBahaei, 2022). The observed neuroanatomical convergence aligns with the substantial genetic similarity, exceeding 90%, between hummingbirds and songbirds, particularly in astrocyte orthologous genes (Aqp4, S100β, Vimentin) and genes related to synaptic plasticity and singing (ZENK, PSD95, Synapsin, Parvalbumin, Doublecortin) (Jarvis et al., 2015). Acknowledging variations in vocal areas and astrocytes among species, it is postulated that the differences may stem from the non-homologous 10% of orthologous genes. This implies that while a portion of the singing learning mechanism is shared, another part evolves independently in response to complexity of communication. While it cannot be definitively concluded that phylogenetic differences explain astrocyte distinctions, the similarity in apprenticeship suggests a relation between astrocyte variations and song complexity at least in house wren. In this species, the abundance and morphological parameters of the astrocytes of the pallium and mesencephalon correlated significantly with the cellular density of the vocal areas involved in complex singing (Supplementary Figure S7). The intricacies observed in astrocytes and vocal areas may be considered a shared vulnerability, emphasizing the intertwined nature of their evolutionary paths.

The difference in the complexity of learned vocalizations between the house wren and the rufous-tailed hummingbird could be related to the cellular density and volume of the vocal areas in the brain. Here, we showed that the brain-to-body weight ratio in the house wren is slightly higher in than the rufous-tailed hummingbird. Also, the RA and HVC were shown to be more prominent in the house wren. It has been found that the size of the vocal repertoire positively correlates with the volume of the HVC and RA in species such as *Cistothorus palustris, Serinus canaria, Acrocephalus schoenobaenus, Taeniopygia guttata,* and *Sturnus vulgaris* (Nottebohm et al., 1981; Kroodsma and Canady, 1985; Nixdorf-Bergweiler, 1996; Airey et al., 2000; Airey and DeVoogd, 2000; Tramontin and Brenowitz, 2000). This indicates that these brain areas are related to vocal complexity, even among males and females of the same species who not engage in duet singing (Nixdorf-Bergweiler, 1996). It has also been observed that the HVC of the zebra finch *Taeniopygia guttata* exhibits neuronal clusters and greater myelination compared to the HVC of the hummingbird *Amazilia* (Gahr, 2000). The HVC, a brain region that integrates the song pathways, a sexually dimorphic trait, contains estrogen receptors, as well as expression of the aromatase enzyme. It should, however, be noted that this does not occur in hummingbirds. These enzymes are activated during the breeding season and are related to the increase in the volume of the vocal brain areas, especially the HVC and RA (Balthazart et al., 1992; Schlinger and Arnold, 1992; Gahr et al., 1993; Gahr and Metzdorf, 1997; Metzdorf et al., 1999; Brenowitz and Larson, 2015; Frankl-Vilches and Gahr, 2018; Larson, 2020).

Although a clear relationship between function and cell density in the vocal areas has not been established, it has been found in the White-browned Sparrow Weaver (*Plocepasser mahali*), a species with a social hierarchy, that the volume and gene expression of HVC and RA, as well as the total number of cells, depending on the social status of males. Dominant males have a larger volume and number of cells in these areas, as well as larger testes. However, this is not reflected in circulating levels of sex hormones (Voigt et al., 2007). Additionally, a relationship has been established between cell size and flight capacity, where smaller cells have a faster metabolism, favoring efficient gas exchange (Gregory, 2018). It can be inferred that the house wren, with its complex song, increases the volume of HVC and RA to facilitate interaction with the syrinx and respiration areas, allowing for more complex vocalization.

We showed that Area X of the house wren had a higher cell density than the rufous-tailed hummingbird. In the canary (*Serinus canaria*), Area X occupies a volume of 2.17 mm^3^, which is much larger than the volume of the RA and HVC (Vellema et al., 2011), suggesting that this structure allows for the incorporation of new repertoires that are regulated by new connections and synaptic plasticity mechanisms like LTP and LTD (Tramontin and Brenowitz, 2000; Ding and Perkel, 2004). Lesions in Area X in adult birds result in changes in the duration and sequence of the song (Kubikova et al., 2014). Being located in the striatum region, an essential part of the basal ganglia, Area X receives glutamate inputs from the pallium and certain regions of the thalamus, such as the medial part of the dorsolateral thalamic nucleus (DLM). It also receives dopaminergic innervation from the mesencephalon. These connections demonstrate the interaction of different brain regions for the song control system to function appropriately (Person et al., 2008). Additionally, Area X has a higher cell density in order to learn songs and maintains the plasticity of the repertoire through more extensive connections in the neural circuits.

We showed that the LMAN of the rufous-tailed hummingbird was more prominent and had higher cell density than the house wren. The LMAN area plays a vital role in vocal learning, although it is not directly involved in song production. Lesions in this area in juveniles reduce vocal learning capacity (Bottjer et al., 1984; Scharff and Nottebohm, 1991; Kittelberger and Mooney, 1999). In *Amazilia amazilia* and *Calypte anna* hummingbirds, it has been observed that the LMAN area exhibits higher cell density compared to the surrounding areas. This area becomes specialized from the early stages of development, enabling juveniles to learn to distinguish the song of their species (Gahr, 2000). For example, in male *Anna’s* hummingbirds, exposure to the tutor’s song induces song learning, demonstrating a process of attention and learning of acoustic stimuli in hummingbirds (Johnson and Clark, 2020). In comparison, in the rufous-tailed hummingbird, there is a strengthening of song learning in the LMAN area, which allows for feedback on songs produced by conspecifics and enables a similarity of vocalizations.

Although astrocytes have been extensively investigated in humans, there still needs to be more understanding of their broader evolutionary nature in other vertebrates. In birds, astrocytes are similar to mammalian astrocytes in structure and function, but there may be differences among species (Verkhratsky et al., 2019; Falcone, 2022). The HVC and the RA contain neuronal cells, glial cells such as astrocytes, ependymal cells, oligodendrocytes, and oligodendrocyte precursor cells (Roberts et al., 2017; Colquitt et al., 2021). Although we did not find robust labeling of astrocytes in the specific vocal areas (LMAN, Area X, RA, and HVC) in the two species, the presence of astrocytes was observed in the pallium, specifically in LEP, and in vascular regions.

We showed abundant astrocytes in like white matter regions, such as the pallidum and mesencephalon, corresponding to the FPL and FRL, respectively. The lateral prosencephalic fascicle (FPL), also known as the medial telencephalic fascicle, is a bundle of nerve fibers that connects the hypothalamus with the limbic system, which is involved in the reward system and basal ganglia (Reiner et al., 2004a; Hernandez et al., 2006; Bradbury and Vehrencamp, 2011; Coenen et al., 2018) It is believed that the FPL plays similar roles in birds, coordinating song production in response to territorial defense and sexual selection, seeking a final reward such as deterring intruders or reproducing. In juvenile zebra finches, *Taeniopygia guttata*, the accuracy of song imitation correlates with brain areas distinct from those in the song motor control system. These areas in the pallium and pallidum, play a role in the early stages of learning before vocal production containing axonal fibers that facilitate connections between various brain regions and the participation of astrocytes, may hold a pivotal role in orchestrating this intricate process (Hamaide et al., 2020). In addition, the reticular formation of the brainstem (FRL) connects the spinal cord and the brain through ascending and descending connections. It plays roles in autonomic, motor, sensory, behavioral, and cognitive functions (Mettler, 1959; Hamaide et al., 2020). Brainstem-spinal pathways are involved in avian locomotion, with predominant locomotor areas found in the ventromedial gigantocellular reticular formation and dorsolateral parvocellular reticular formation (Steeves et al., 1987). These findings have been observed in other vertebrates (Steeves and Jordan, 1980; Eidelberg et al., 1981).

We found GFAP astrocytes in the LEP in the house wren and rufous-tailed hummingbird, also in the FPL and FRL in the house wren, and S100β astrocytes in the same three regions in the hummingbird. These areas are mainly composed of white matter and show strong myelination (Karten et al., 2013; Gedman et al., 2021). The presence of myelin in the brain and spinal cord areas suggests a need for fast and efficient communication between brain regions through the pathways passing through the FPL and FRL (Nickel and Gu, 2018). Myelination in these pathways is crucial for the precise control of bird vocalization. During development, differences in the speed and degree of myelination are observed in the vocal areas, with the DLM region showing early myelination and the HVC exhibiting a slower process (Champoux et al., 2021). Bird vocalization relies on the basal ganglia circuits, a region that integrates different brain areas (Creese and Iversen, 1975). Both neurons and astrocytes play a role in regulating dopamine levels in the basal ganglia (Smith and Kieval, 2000; Björklund and Dunnett, 2007; Rice et al., 2011; Vaughan and Foster, 2013). A lack of myelination can lead to disorders in motor coordination, cognition, and speech in humans (Duncan and Radcliff, 2016).

Comparative studies of astrocytes are based on cellular morphology. The GFAP protein is a house marker used to detect mature astrocytes, but it does not represent the entire heterogeneity of the astrocyte population. Immunostaining for GFAP allows visualization of astrocyte morphology (Hol and Pekny, 2015; Yang and Wang, 2015; O’Leary and Mechawar, 2021; Falcone, 2022). Additionally, the S100β protein is mainly found in astrocytes and is used as a marker in neurological diseases (Michetti et al., 2019). This contributes to understanding the presence and function of astrocytes in regions where they are expressed. Astrocyte morphology can vary between species, such as fish, amphibians, reptiles, and birds. GFAP is observed around the ventricles in thick and straight bundles in fish (Chen et al., 2020). In amphibians, astrocytes do not exhibit the typical star-shaped form (Onteniente et al., 1983). In reptiles, cells with clear astrocyte morphology are found (Bodega et al., 1990). In birds, astrocytes have similar functions to mammals, surrounding neuronal synapses and contacting blood vessels (Bairati and Bartoli, 1955; King, 1966).

The shape of astrocytes in birds varies according to the species, with different numbers and locations (Bairati and Bartoli, 1955; Falcone, 2022). In humans, protoplasmic astrocytes are mainly found in the brain’s gray matter, such as the cerebral cortex. They have large, ramified cell bodies with multiple short and dense processes extending in different directions, involved in contact with neurons and synapses (Bushong et al., 2002). On the other hand, fibrous astrocytes are housed in the white matter of the brain and have elongated and thin cell bodies, with long and slender processes predominantly in one direction. These processes provide structural and metabolic support along the nerve pathways (Miller and Raff, 1984; Oberheim et al., 2009, 2012; Sofroniew and Vinters, 2010; Tabata, 2015). In both species of birds, GFAP astrocytes located in the LEP had a morphology like protoplasmic astrocytes, while in house wren the FPL and FRL, astrocytes showed a morphology similar to fibrous astrocytes; this arrangement allows the bird to hear and perceive its environment, as well as respond appropriately to auditory stimuli. In the rufous-tailed hummingbird, S100β astrocytes have a simpler morphology, resembling varicosities or processes with a barely apparent soma from the mesencephalic areas to the vocal areas. Based on the heterogeneity of astrocyte populations, we recommend extending the analysis using astrocyte markers, such as GS and EAAT, which possibly link synaptic activity in vocal brain areas.

In conclusion, this comparative study provides valuable information on differences in vocal areas and astrocytes in two species of vocal learning birds, which suggests specific specialization in each species that would support differences in song complexity in these species. Despite not finding a typical astrocytic morphology in vocal areas, the GFAP astrocytes were located in motor relay areas necessary to support vocal production and complexity. These findings allow us to propose new ecophysiological studies in which incorporating other phylogenetically separated species and more individuals of each species could reproduce the associations between astrocytes and vocal behavior.

## Data availability statement

The original contributions presented in the study are included in the article/Supplementary material, further inquiries can be directed to the corresponding author.

## Ethics statement

The collection of biological specimens is covered by the framework permit for the collection of wild specimens for non-commercial purposes, which was issued by ANLA in resolution 1461 of December 3, 2014. The size sampling permission and the procedure was approved by the Ethics Committee for Animal Experimentation at the University (CEEA), resolution 139 of March 29, 2021.

## Author contributions

CL-M: Data curation, Formal analysis, Investigation, Methodology, Software, Validation, Writing – original draft, Writing – review & editing. SH-M: Data curation, Formal analysis, Investigation, Methodology, Software, Writing – review & editing. PH: Data curation, Formal analysis, Investigation, Methodology, Software, Writing – review & editing. JT: Data curation, Formal analysis, Investigation, Methodology, Software, Writing – review & editing. GC-G: Data curation, Formal analysis, Investigation, Methodology, Software, Writing – review & editing. HR-G: Conceptualization, Funding acquisition, Investigation, Supervision, Writing – review & editing, Methodology. RP-D: Conceptualization, Formal analysis, Funding acquisition, Investigation, Methodology, Project administration, Supervision, Writing – original draft, Writing – review & editing.

## Figures and Tables

**Table:** 

AFP	Anterior forebrain pathway
At	*Amazilia tzacatl*
Av	Avalanche core
CM	Caudal mesopalium
CNS	Central nervous system
DLM	Medial part of the dorsolateral thalamic nucleus
FPL	Lateral prosencephalic fasciculus
FRL	Lateral reticular formation of the midbrain
GFAP	Glial fibrillary acidic protein
HVC	High vocal center
LEP	Laminar edge of pallium
LMAN	Lateral magnocellular nucleus of the anterior nidopallium
NCM	Caudomedial nidopallium
Nif	Kernel interface
nXIIts	Tracheosyringeal portion of the hypoglossal nucleus
RA	Robust nucleus of arcopallium
SMP	Song motor pathway
Ta	*Troglodytes aedon*
OV	Nucleus ovoidalis
Uva	Nucleus uvaeformis
VRG	Ventral respiratory group
