# Supplementary figures and images for: Differences in vocal brain areas and astrocytes between the house wren and the rufous-tailed hummingbird
Source: Front Neuroanat. 2024 Mar 27;18:1339308. doi: 10.3389/fnana.2024.1339308 (PMC11004282; doi:10.3389/fnana.2024.1339308)

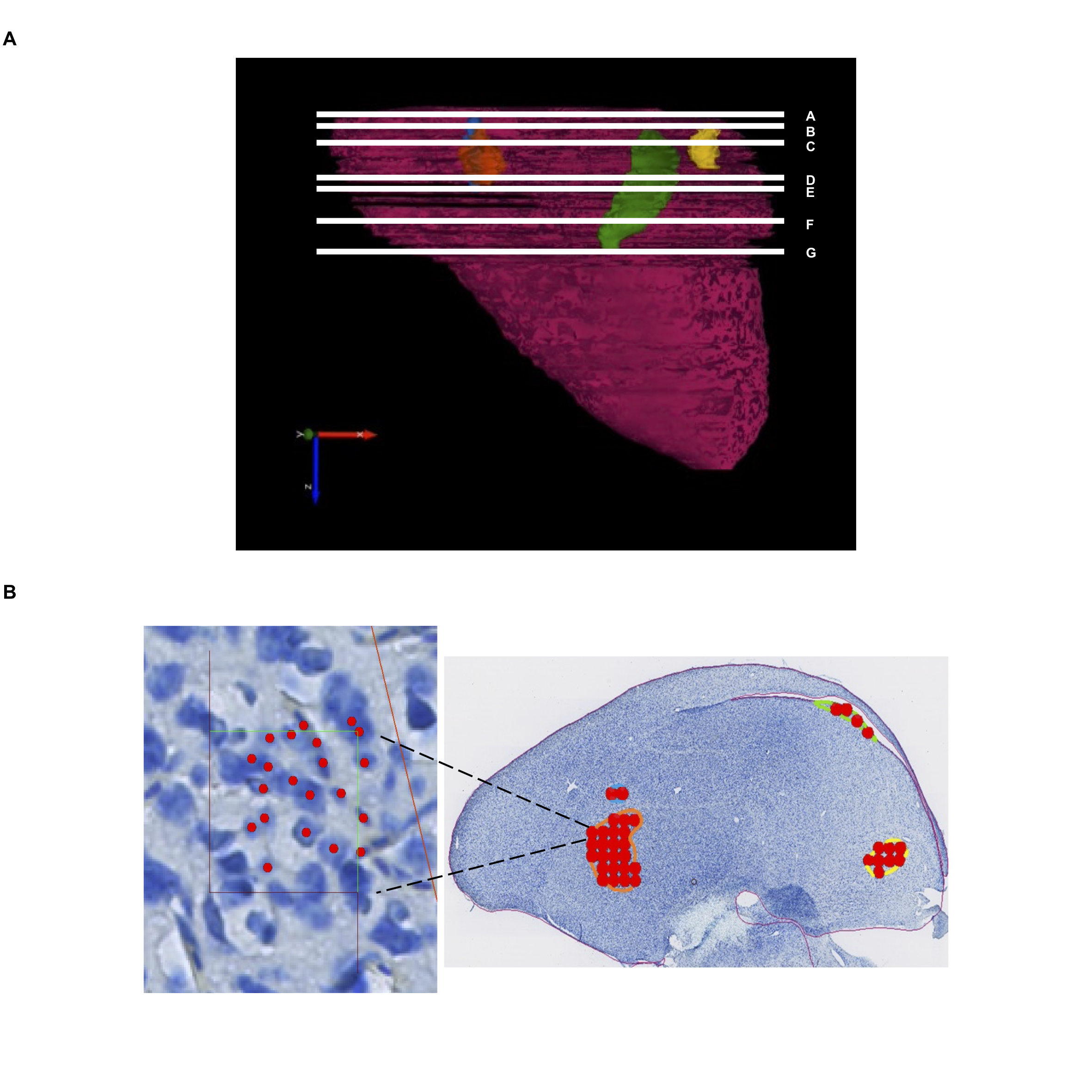

Supplement: Supplementary file 1 [file Image_1.TIFF]

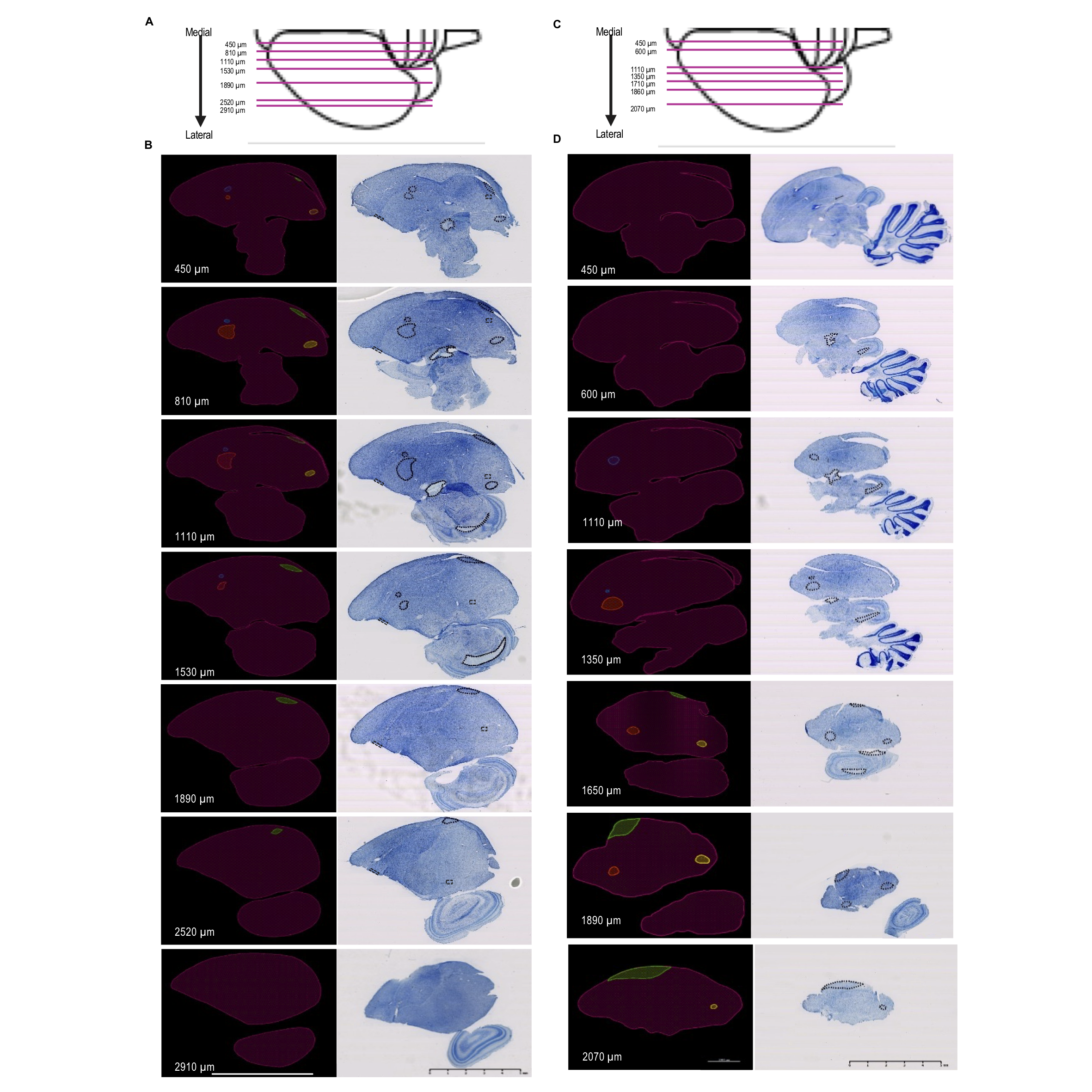

Supplement: Supplementary file 2 [file Image_2.TIFF]

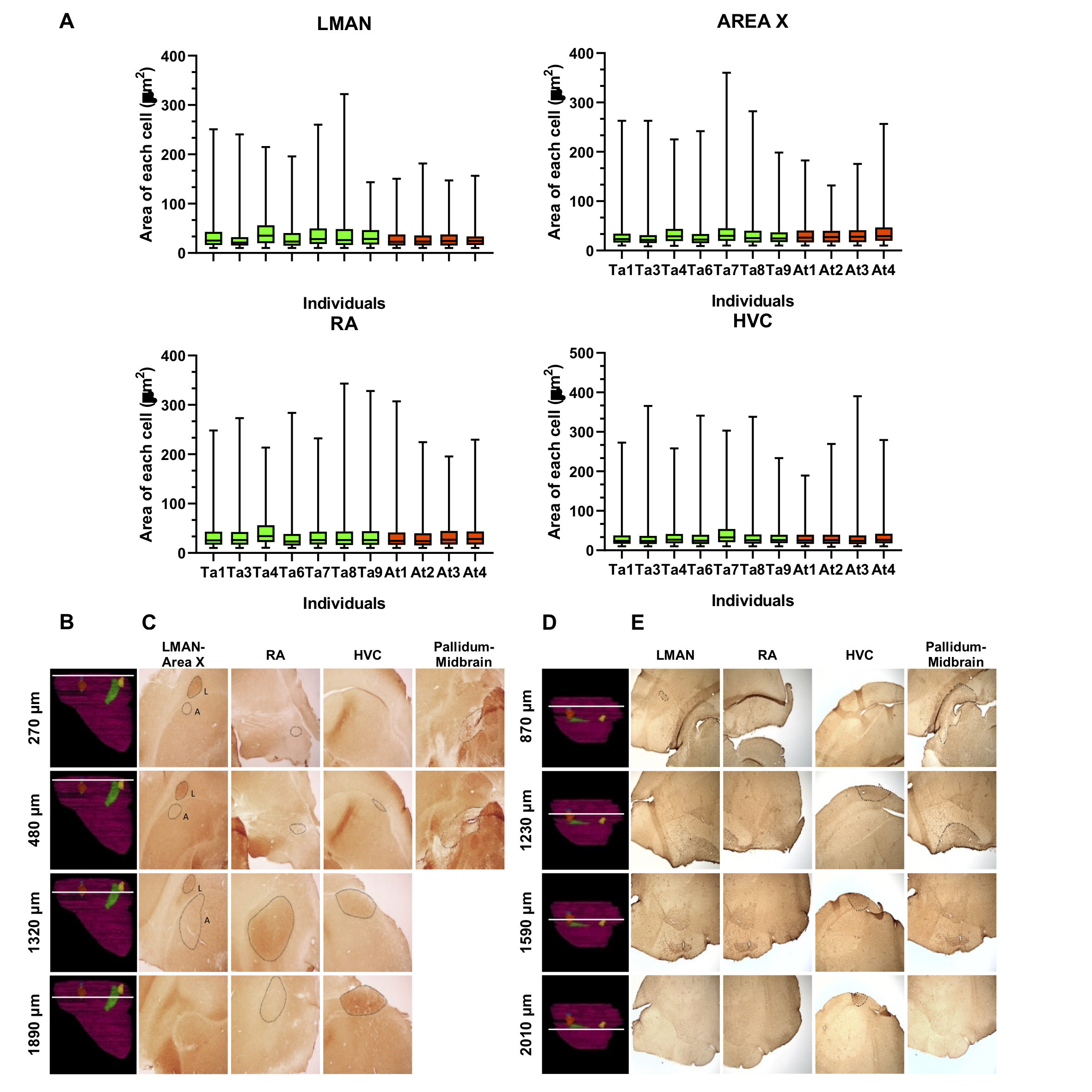

Supplement: Supplementary file 3 [file Image_3.TIFF]

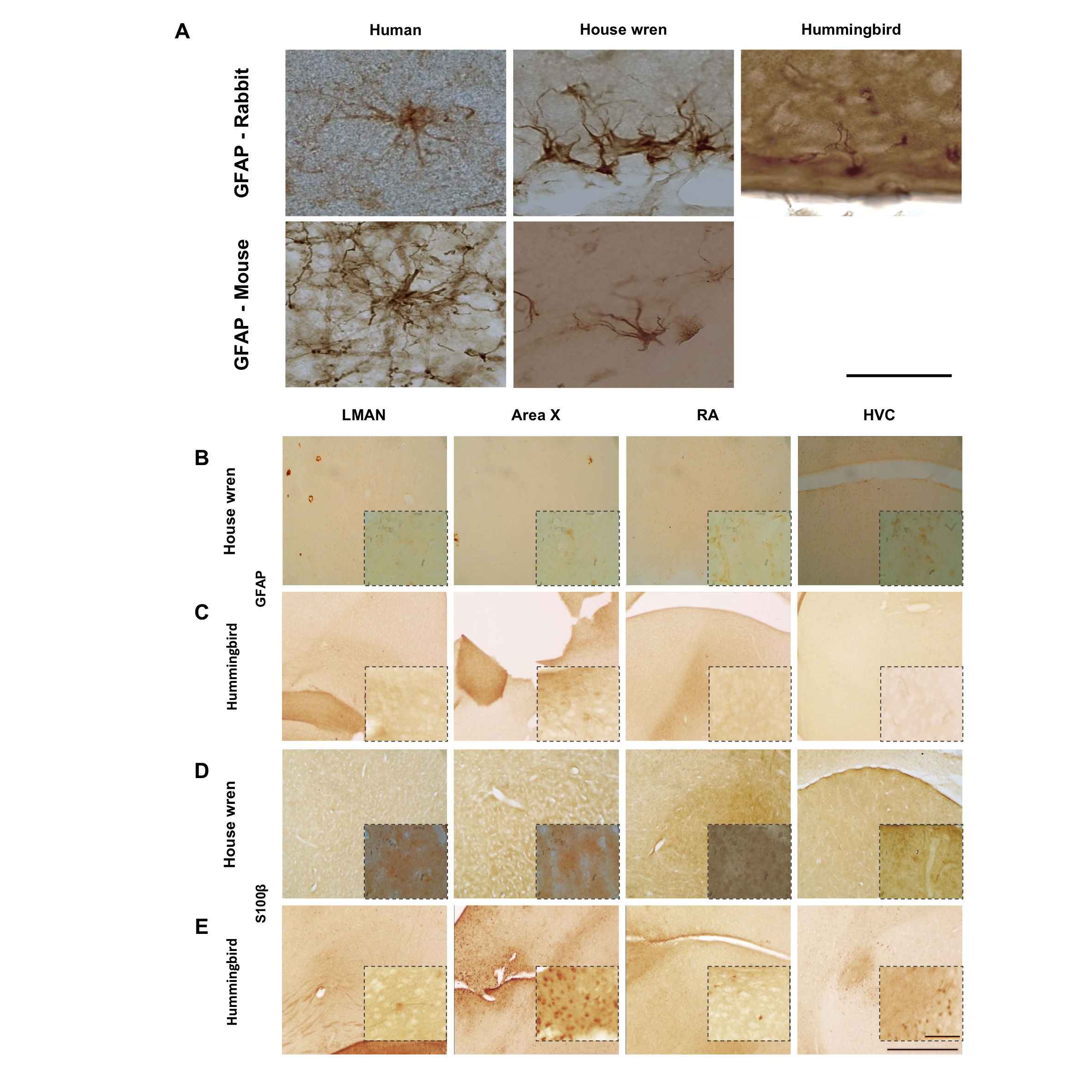

Supplement: Supplementary file 4 [file Image_4.TIFF]

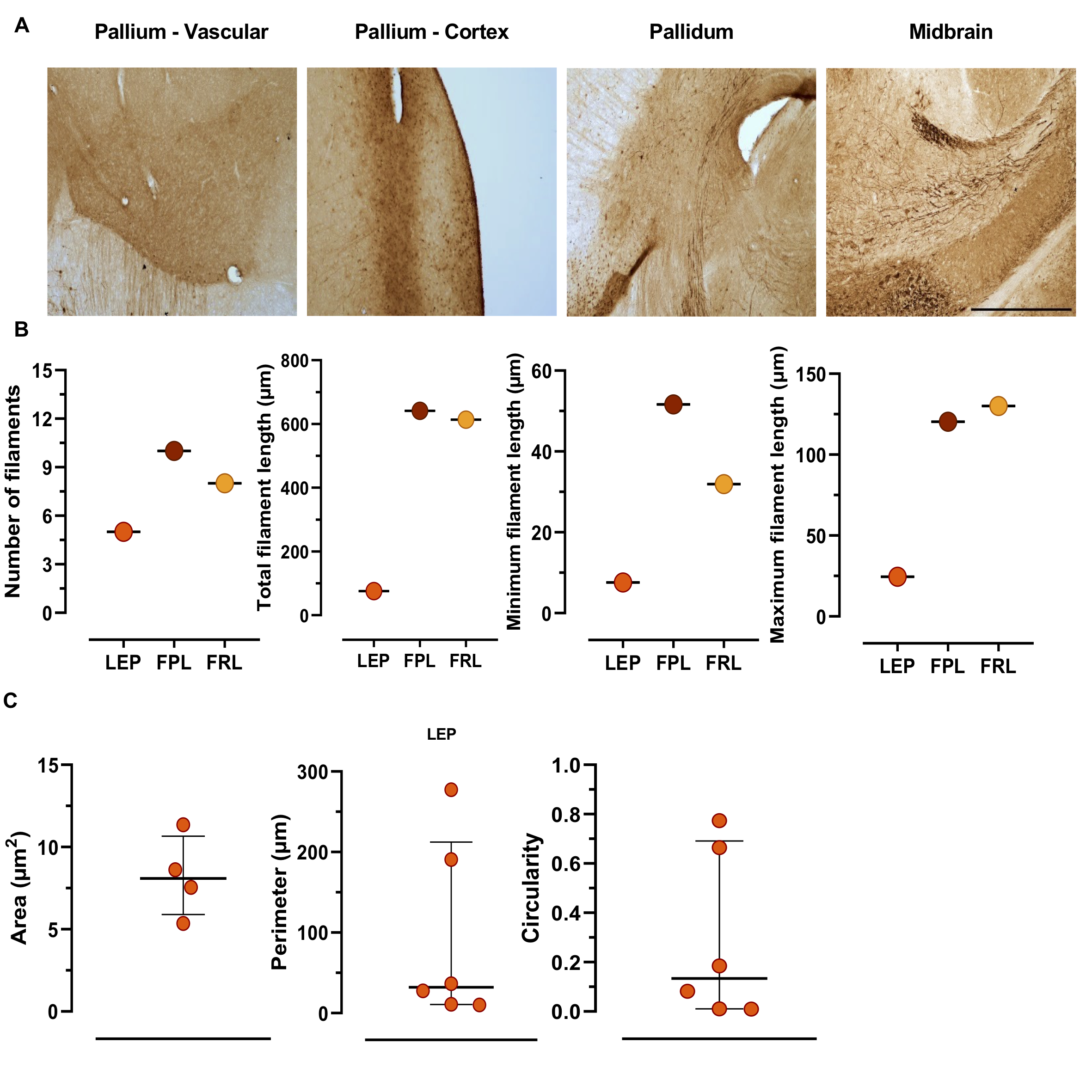

Supplement: Supplementary file 5 [file Image_5.TIFF]

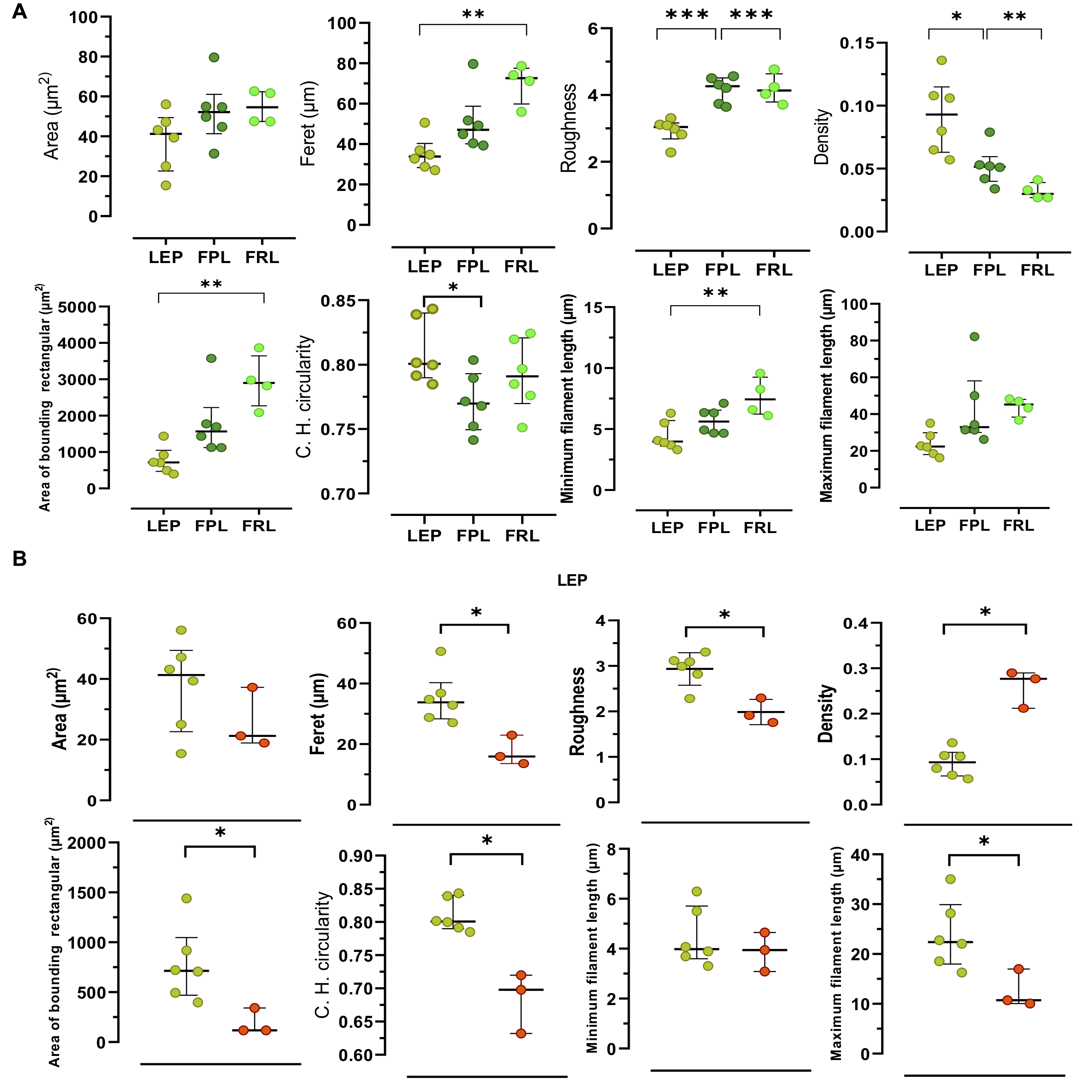

Supplement: Supplementary file 6 [file Image_6.TIFF]

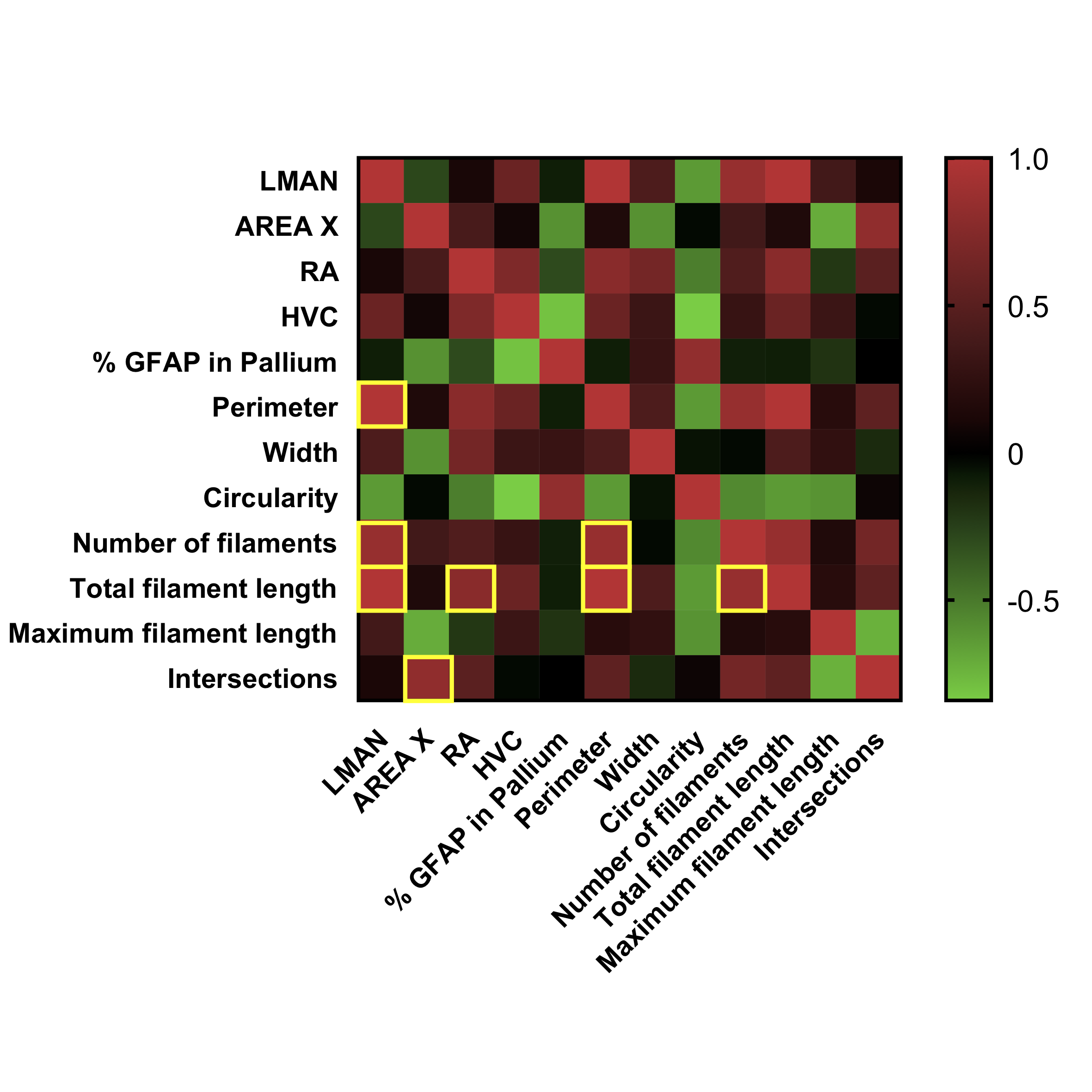

Supplement: Supplementary file 7 [file Image_7.TIFF]
